# Effect of nature on the mental health and well-being of children and adolescents: meta-review

**DOI:** 10.1192/bjp.2024.109

**Published:** 2024-09

**Authors:** Tessa Lomax, Joseph Butler, Andrea Cipriani, Ilina Singh

**Affiliations:** Department of Psychiatry, University of Oxford, Oxford, UK; and Oxford Health NHS Foundation Trust, Warneford Hospital, Oxford, UK; Oxford Health NHS Foundation Trust, Warneford Hospital, Oxford, UK; Department of Psychiatry, University of Oxford, Oxford, UK; Oxford Health NHS Foundation Trust, Warneford Hospital, Oxford, UK; and Oxford Precision Psychiatry Lab, NIHR Oxford Health Biomedical Research Centre, Oxford, UK; Department of Psychiatry, University of Oxford, Oxford, UK

**Keywords:** Childhood experience, depressive disorders, low- and middle-income countries, neurodevelopmental disorders, psychosocial interventions

## Abstract

**Background:**

Urbanisation is taking place worldwide and rates of mental illness are rising. There has been increasing interest in ‘nature’ and how it may benefit mental health and well-being.

**Aims:**

To understand how the literature defines nature; what the characteristics of the nature intervention are; what mental health and well-being outcomes are being measured; and what the evidence shows, in regard to how nature affects the mental health and well-being of children and adolescents.

**Method:**

A meta-review was conducted, searching three databases for relevant primary and secondary studies, using key search terms including ‘nature’ and ‘mental health’ and ‘mental well-being’. Inclusion criteria included published English-language studies on the child and adolescent population. Authors identified the highest quality evidence from studies meeting the inclusion criteria. Data were extracted and analysed using descriptive content analysis.

**Results:**

Sixteen systematic reviews, two scoping reviews and five good quality cohort studies were included. ‘Nature’ was conceptualised along a continuum (the ‘nature research framework’) into three categories: a human-designed environment with natural elements; a human-designed natural environment; and a natural environment. The nature ‘intervention’ falls into three areas (the ‘nature intervention framework’): access, exposure and engagement with nature, with quantity and quality of nature relevant to all areas. Mental health and well-being outcomes fit along a continuum, with ‘disorder’ at one end and ‘well-being’ at the other. Nature appears to have a beneficial effect, but we cannot be certain of this.

**Conclusions:**

Nature appears to have a beneficial effect on mental health and well-being of children and adolescents. Evidence is lacking on clinical populations, ethnically diverse populations and populations in low- and middle-income countries. Our results should be interpreted considering the limitations of the included studies and confidence in findings.

The prevalence of mental health diagnoses is rising globally, with up to 20% of the world's adolescent population estimated to have a mental health condition.^1^ This is mirrored in the UK, with estimated rates of probable mental disorder in the 7- to 16-year-old population rising from 12% in 2017 to 18% in 2022.^2^ One posited factor in this increase is urbanisation.^3^ By 2050, 68% of the world's population is expected to live in urban areas,^4^ and questions have been raised about how a lack of exposure or connection to nature, coined ‘nature-deficit disorder’,^5^ may play a role. Children and adolescents are spending more time indoors using screens, with concerns raised that both reduced time outdoors in nature and more screen time may lead to unfavourable psychological outcomes.^6^

Current research suggests nature may be beneficial for children's brain and cognitive development,^7^ as evidenced by three-dimensional magnetic resonance imaging and cognitive testing, with specific improvements in working memory and attention.^8,9^ Current research also indicates its beneficial effect on mental health and well-being, including reducing stress,^10,11^ improving attention-deficit hyperactivity disorder (ADHD) symptoms,^10–13^ reducing depressive symptoms and psychological distress^13^ and fostering emotional well-being.^10–13^ Many different theories have been put forward to explain the proposed positive effects of nature on child and adolescent mental health.^8,14–19^ One such theory is the biophilia hypothesis, which puts forward that humans are drawn to and have an important evolutional bond with nature.^8,17^ In this vein, it has been postulated that contact with nature is crucial for brain development.^8^ The other key theory, attention restoration theory, puts forward that exposure to natural environments leads to improved cognitive performance by promoting restoration of a limited cognitive resource – directed attention.^19^ Other mechanisms proposed include increasing physical activity, increasing social contact, reducing stress^14,18^ (with the associated stress reduction theory^20^), mitigating against the effects of harmful environmental exposures (e.g. air and noise pollution),^14,16^ increasing exposure to beneficial environmental exposures (e.g. plant phytoncides)^15^ and improving immune system functioning^14,15^ and sleep.^15^ Green space for children is thought to enhance cognitive function, not only by some of the above-mentioned mechanisms but also by promoting engagement, risk taking, discovery, creativity and a sense of wonder, strengthening the sense of self and enhancing psychological restoration.^16^

Nature prescriptions or ‘green social prescribing’, whereby a health or social professional ‘prescribes’ time outdoors in nature to patients informally or formally (via organised programmes), have gained popularity in recent times.^21^ This may in part be driven by the UK government's Green Social Prescribing Programme, launched in 2021 to embed green social prescribing in mental healthcare,^22^ as well as green social prescribing being seen as a sustainable form of social prescribing.^21^ In a recent systematic review and meta-analysis examining the effect of nature prescriptions on cardiometabolic and mental health and physical activity, such prescriptions have been shown to have a moderate to large effect on improving depression and anxiety scores; however, research is largely focused on adult populations,^21^ making our understanding of how they may benefit children and adolescent's mental health less certain.

There have been attempts to synthesise the current evidence on the effect of nature on child and adolescent mental health and well-being. Recent systematic and scoping reviews indicate that there is a lack of consistency on how ‘nature’ and ‘mental health’ and connected terms, such as ‘well-being’, are defined and investigated.^23^ For example, some reviews considered exposure to ‘green space’ (e.g. walking in a green park) as a proxy for nature,^9,12^ whereas others examined nature-based activities (e.g. gardening) where people were directly engaging with nature.^11^ It is standard practice in the current literature to conflate ‘mental health’ and ‘well-being’, although these could also be conceptualised and operationalised as distinct entities.

In general, there is consensus that, even if ‘nature’ appears to be good for mental health and well-being, further evidence is needed to draw firm conclusions.^11–13^ In this meta-review we aimed to summarise the best quality data to help identify gaps and draw further conclusions, from collating bigger bodies of evidence where appropriate. By clarifying key concepts and definitions of nature and exploring how nature, mental health and well-being, and their interactions, are being examined, we also developed a conceptual framework that can be used and taken forward to help give direction and clarity to this field of research for future studies.

## Method

This review is built around four key questions:

- How does the literature define ‘nature’?
- What are the characteristics of the nature intervention?
- What are the mental health and well-being outcomes being measured?
- What does the evidence show with regard to how nature affects the mental health and well-being of children and adolescents?

The study protocol was registered on Open Science Framework (https://osf.io/ehyc8/). As this is a meta-review of existing published literature, ethical approval was not required.

### Search strategy

The full search strategy can be found in the Supplementary material available online at https://doi.org/10.1192/bjp.2024.109. The search included terms relating to nature, mental health and well-being, and children and adolescents. Search terms used relating to nature include (but are not limited to): Natural environment, natural world, nature, forests, grasslands, wetlands, wilderness, wilderness experience, horticulture therapy, gardens, gardening, parks, green space, blue space, outdoor*, ecotherap* and nature-based. Mental health and well-being outcomes used include (but are not limited to): mental health, mental disease, mental wellbeing, psychological well-being, well-being, cognitive development, infant development, depression, anxiety, child psychiatry, adolescent psychiatry. The following databases were searched: MEDLINE, Embase and PsycINFO. Searches were limited to the English language and from 2010 until 31 October 2022. The search was from 2010 onwards, as an initial search using the MEDLINE search strategy found little evidence on nature published before then (Supplementary Figs 2 and 3).

### Inclusion criteria

Studies with children and/or adolescents up to the age of 18 were included, as well as studies using a sample population up to 25 years of age that had a mean age below 18. Studies also including adults were included if there was a subgroup analysis on our population of interest (<18 years). The exposure/intervention of interest was ‘nature’. We took a broad inclusive view of the definition of ‘nature’ (thus including studies looking at surrounding green space to those looking at wilderness therapy). Outcomes included any measure of mental health and well-being (e.g. developmental, cognitive and behavioural outcomes), using validated mental health and well-being scales as well as less well-defined measures, such as self-perceived stress.

### Study type and source of evidence selection

Both primary (observational or experimental) and secondary (reviews with or without quantitative synthesis) studies were included. Unpublished studies, such as dissertations, were excluded. Two reviewers independently undertook title and abstract screening, then full-text examination. Any disagreements were settled between the two reviewers, seeking advice from a third reviewer in case of discrepancies. After identifying all systematic and scoping reviews, the reviews were examined to ascertain whether any individual studies were not included in the retrieved evidence base (i.e. primary studies that were published after the date of the last identified review). Review authors were contacted for missing information, as appropriate.

### Certainty of evidence

Included studies were critically appraised according to their study type, using the following validated tools: the AMSTAR 2 tool for systematic reviews; RoB 2 (version 2 of the Cochrane risk-of-bias tool) for randomised controlled trials; ROBINS-I (Risk Of Bias In Non-randomised Studies – of Intervention) for experimental non-randomised studies; and the Newcastle–Ottawa Scale (NOS) for observational studies. The NOS tool was adapted for cross-sectional and cohort studies respectively to align with our study aims, considering the following specific confounders relevant to the literature base: pollution, noise, social contact/engagement and exercise (Supplementary material). All information about the assessment tools can be found in our protocol (https://osf.io/ehyc8/).

### Data extraction

Two independent reviewers extracted data on: author, year of publication, journal (impact factor); country of origin; aims/purpose; population characteristics; methodology; how nature is defined; nature intervention characteristics; mental health and well-being outcome measures; key findings; any mechanisms. Data extraction was first performed on 15 November 2022.

### Analysis of the evidence

Data were analysed using a descriptive content analysis, broken down into the four questions that reflect the meta-review's aims (see above).

## Results

### Study selection

We retrieved 1657 articles in total, after removal of duplicates. After title, abstract and full-text screening, 162 studies were eligible for inclusion (the PRISMA flowchart appears in Supplementary Fig. 1). From these, we identified the evidence with the highest degree of certainty – 16 systematic and 2 scoping reviews, as well as 5 cohort studies (published after the date of the last identified review). The Supplementary material lists the 139 studies that were potentially eligible but not included in this meta-review owing to selection for inclusion only of the highest quality evidence to answer the study aims, as per protocol.

**Fig. 1:**
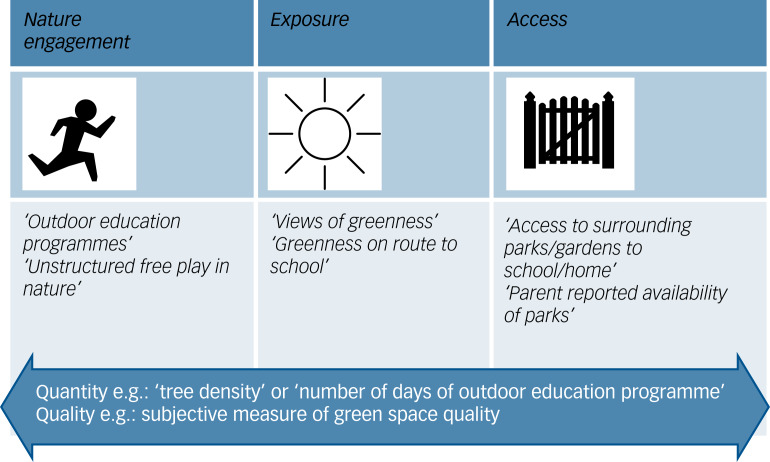
The ‘nature intervention framework’: framework when examining the nature intervention and examples of how current evidence (from included studies) fits the proposed framework.

### Study characteristics

See Supplementary Table 1 for the study characteristics of included studies and key findings.

All reviews were from institutions in high- and middle-income countries. Two reviews^10,24^ did not give information on included studies’ country or continent of origin. Five reviews reported some detail on continent or country of origin of included studies but did not provide detail on country of origin for all studies.^12,25–28^ Of the 441 primary studies where country of origin was reported (identified from reviews and the 5 cohort studies), studies were almost exclusively from high-income countries (HICs) (*n* = 431), with only 9 studies coming from upper middle-income countries (South Africa, *n* = 5; Turkey, *n* = 2; Bulgaria, *n* = 1; Jamaica and St Vincent, *n* = 1) and one study from a low-income country (Iran). Of the primary studies where country of origin was reported, the majority (*n* = 418) came from HICs, most likely examining majority White populations.^29–31^

In general, ethnicity was poorly explored, with seven reviews^10,11,24,32–35^ not reporting on whether included studies adjusted or collected data on ethnicity. Of the reviews that did report this, ethnicity was rarely adjusted for in included studies (Supplementary Table 1). Only one^36^ out of the five cohort studies included in this meta-review adjusted for ethnicity and this was in a limited way (Hispanic versus non-Hispanic). Socioeconomic status (SES) was more routinely collected and adjusted for in primary studies compared with ethnicity, but reporting on SES in reviews was generally poor, with eight reviews not reporting on whether included studies collected data on and adjusted for SES (Supplementary Table 1).^10,11,24,27,29,32,34,35^

### Certainty of evidence

Only five primary studies (all cohort studies) published after the date of the last identified review were found to be of good quality and are included in this review (Supplementary material). In two of these five studies, out of eight risk-of-bias domains, high risk of bias was identified only in the selection domain (demonstrating that the outcome of interest was not present at the start of the study) and in the outcome domain (relating to adequacy of follow-up).^36,37^ In the remaining three studies^39,40,42^ high risk of bias was only identified in the selection domain (demonstrating that the outcome of interest was not present at the start of the study). These five primary studies were identified after reviewing all cross-sectional, cohort and experimental studies after the date of the last systematic review and examining their quality. There was cause for concern about the risk of bias in all systematic and scoping reviews in various domains, with high risk of bias identified in more than half of reviews in the following domains: reporting *a priori* protocol (with justifications of deviations from the protocol); using a comprehensive literature search strategy (although the majority of reviews were marked down for giving no justification of the English-language limitation); data extraction performed in duplicate; providing a list of excluded studies with justifications for exclusions; and reporting on the source of funding of the included studies (see Supplementary material).

### How does the literature define ‘nature’?

Results from the descriptive content analysis reveal that ‘nature’ appears to be defined or conceptualised along a continuum into three broad categories: a human-designed environment with natural elements (e.g. outdoor tarmacked playground with surrounding trees); a human-designed natural environment (e.g. garden); or a natural environment (e.g. woodland). Some studies found in this review can be categorised using this framework,^10,32,35^ but many articles did not clearly define what they meant by ‘nature’^13,24,26,33,38–40^ or included heterogeneous studies where nature appeared to be conceptualised in different ways^11,28–31,34,37,41^ (Supplementary Table 2). Many studies referred to more general concepts, such as ‘green space’ or ‘greenness’.^12,25,27,36,39,42^ Such cases could not be categorised without making unfounded assumptions.

### What are the characteristics of the nature intervention?

Results from the descriptive content analysis show that the nature ‘intervention’ falls into three key areas: access to nature, exposure to nature or engagement with nature; the quantity and quality of nature are relevant to all three areas (Fig. 1). Tillman et al's framework^11^ also included the three areas (access, exposure or engagement), but it did not include quantity and quality of nature. Research studies examined access, exposure or engagement with nature (or a mixture of these). All five cohort studies reported that they were looking at green space exposure, but they were actually using proxy measures of exposure (i.e. presence of ‘surrounding green space’ near the home (*n* = 5) or school (*n* = 1)) and in two cohort studies they also examined accessibility to green space.^37,42^ Five reviews^12,26–28,33^ reported that they were examining green space or ‘greenness’ exposure, but they all included studies in their review that looked at proxy measures of exposure (surrounding green space) and/or studies that looked at other things, such as access to green space^26^ or activities in green space.^33^ Some studies made explicit the quantity (e.g. 1 day a week in a forest school); however, very few considered the ‘quality’ of the nature intervention. Fig. 1 gives examples of how studies may fit this framework.

### What are the mental health and well-being outcomes being measured?

**Fig. 2:**
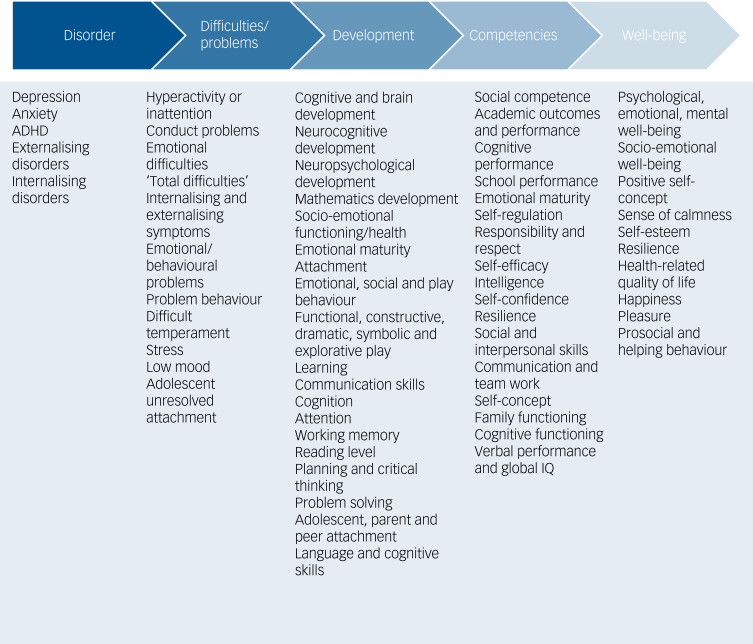
Mental health and well-being outcomes identified in the meta-review exist along a continuum, with disorder at one end and well-being at the other. Outcomes measured by the included studies are detailed below the continuum. ADHD, attention-deficit hyperactivity disorder; IQ, intelligence quotient.

Mental health and well-being outcomes fit along a continuum, with ‘disorder’ at one end and ‘well-being’ at the other. Fig. 2 shows the range of mental health and well-being outcomes found in the meta-review.

The majority of studies examined symptoms or behaviours relating to mental health and well-being; a few looked at mental disorders, namely anxiety, depression and ADHD.^11,25–27,36^ No studies looked at bipolar affective disorder, psychosis, autism spectrum disorder (ASD) or eating disorders. Cognitive and developmental outcomes were examined frequently,^10,24,26,29–31,33–35,37,40,42^ perhaps because childhood and adolescence is a key period of brain development. Studies focused on younger child populations tended to focus more on development and cognitive outcomes, whereas studies focused on older populations (adolescents) tended to focus more on traditional mental health outcomes.

### What does the evidence show?

Nature appears to have a beneficial effect on mental health and well-being, with largely significant positive results (indicating a beneficial effect) or positive results with unknown significance. Supplementary Table 2 presents summary results, highlighting studies supporting nature's beneficial effect, studies where the evidence was unclear and studies indicating that nature had a negative effect. It also details how authors defined and examined ‘nature’ and how this may fit the proposed frameworks. Outcomes rated as unclear in Supplementary Table 2 are further described in the table, giving context. There were no studies/reviews indicating that nature had a negative effect.

#### Children only

In general, the evidence was in support of nature's beneficial effects on children's mental health-related outcomes; however, no review or cohort study focused on well-being outcomes in children. All reviews were in support of nature's beneficial effect on mental health; however, the three cohort studies were more mixed, with one indicating benefit, one having a mixture of beneficial and unclear results^37^ and the third having unclear results^36^ (Supplementary Table 2). Some reviews examined only observational evidence, assessing the association between green space exposure and mental health outcomes,^26,33,38^ and one focused on ‘nature play’, an experimental nature intervention.^29^ Some reviews examined cognitive or neuropsychological developmental outcomes^26,29,33^ and others mental health difficulties and emotional outcomes.^29,38^

#### Adolescents only

All four reviews^13,25,27,32^ found support for nature's beneficial effect, except for the reviews by Reece et al^27^ (where the evidence was uncertain) and Fleckney & Bentley^25^ (when examining longitudinal evidence) (Supplementary Table 2). All four reviews assessed a measure of mental health and/or mental well-being. Zhang et al^13^ assessed mental well-being outcomes; Fleckney & Bentley^25^ assessed mental health and well-being outcomes; Reece et al^27^ focused on anxiety and depression; and Fang et al^32^ looked at self-efficacy. Similar to reviews assessing the child population, reviews involving adolescents focused primarily on assessing observational evidence of green space/nature (*n* = 3).^13,25,27^ Only one review^32^ focused on a form of nature engagement (outdoor education programmes).

#### Both children and adolescents (under-18s)

All ten reviews that focused on both child and adolescent populations (under-18s) supported nature's beneficial effects on mental health and well-being. However, evidence was less clear for some outcomes in two reviews (Supplementary Table 2), i.e. self-concept, problem-solving and mood^35^ and emotional well-being, self-esteem and depression.^11^ Of the two cohort studies, one^39^ supported nature's beneficial effects in improving mental well-being; in the other,^40^ the evidence for beneficial effects of nature on childhood development and academic outcomes was less clear, as even though the direction of change was in support of nature's beneficial effects, results did not reach significance.

A subset of reviews assessed both observational and experimental evidence;^11,24^ another subset focused on experimental evidence, looking at the following forms of nature engagement: ‘immersive nature experience’;^35^ ‘family-based outdoor treatment’;^30^ ‘nature-specific outdoor learning’;^34^ or ‘school yard greening’.^41^ The remaining four reviews focused on observational evidence assessing green space exposure.^10,12,28,31^ One review^30^ focused on clinical populations, whereas the remainder focused largely on non-clinical general populations. Seven reviews^10–12,24,34,35,41^ assessed for mental health and well-being outcomes; one^30^ assessed for mental health problems; three reviews assessed for developmental outcomes,^12,31,34^ two assessed for behavioural outcomes^24,28^ and one assessed for academic outcomes.^34^

## Discussion

In this meta-review we found that ‘nature’ is conceptualised along a continuum into three broad categories: a human-designed environment with natural elements; a human-designed natural environment; and a natural environment. We call this continuum the ‘nature research framework’. Nature ‘interventions’ fall into three key areas – access, exposure, or engagement with nature – with quantity and quality of nature being relevant to all three areas (Fig. 1). We call this the ‘nature intervention framework’. Mental health and well-being outcomes fit along a continuum, with ‘disorder’ at one end and ‘well-being’ at the other. Following the use of validated critical appraisal tools, we found medium certainty of evidence with medium to high risk of bias in reviews and recent cross-sectional and non-randomised interventional studies, and lower risk of bias in recent cohort studies (Supplementary material).

We judge that in general (i.e. non-clinical) child and adolescent populations, there is sufficient indication of benefit to motivate more high-quality research into the relationship between nature and mental health and well-being (including cognitive and academic outcomes). The evidence is more uncertain in child and adolescent clinical mental health populations owing to the small number of studies. ADHD has received the most research attention, although the majority of studies examining ADHD symptomatology, such as inattention and hyperactivity, were conducted in non-clinical populations. This research focus on ADHD and inattention may be partly driven by having a well-established underpinning mechanistic theory – attention restoration theory. More high-quality studies in different clinical groups with different diagnoses are needed to understand whether nature interventions have benefits for such groups. There is a binary approach when diagnosing mental disorder verses mental health; however, we know that in fact mental health and mental disorder sit along on a continuum.^43^ Therefore, it is likely that, for a subset of clinically diagnosed children and adolescents, there will be benefits similar to those found in non-clinical groups.

### Sociodemographic characteristics

The lack of data on other sociodemographic factors, particularly race/ethnicity, severely limits the generalisability of the findings and undermines research commitments to equality, diversity and inclusion (EDI). Despite the majority of studies including a measure of SES, there needs to be a better exploration of how SES mediates nature's effect through more detailed comparison across socioeconomic groups and more consistent data collection on both household and neighbourhood socioeconomic measures. Alderton et al^38^ found only three studies comparing findings across socioeconomic groups, with two of these finding greater benefits in those from poorer socioeconomic backgrounds (also supported by Zhang et al^13^), suggesting that those from poorer socioeconomic groups may benefit more.

### Defining nature

Our nature research framework enables better testing of two prominent hypotheses regarding the mechanisms driving the relationships between nature interventions and mental health and well-being benefits. Attention restoration theory posits that the natural environment is particularly restorative to our attention.^44^ Stress reduction theory hypothesises that the natural environment reduces stress and promotes parasympathetic activity.^20^ However, there is little disaggregation of what ‘natural environment’ means operationally; how natural the environment must be; or how much of it (dose) one needs to gain benefit. It is pressing that we understand these things, as with global urbanisation, people's ‘nature’ will most likely come from ‘human-designed environments with natural elements’ rather than wholly natural environments. It is important to know whether we can still see beneficial effects of nature using our urban parks, or whether more natural environments within urban settings are required to harvest nature's protective effects.

Some evidence is emerging, mostly from the adult literature, highlighting how the type and dose of nature may affect outcomes, such as nature connectedness (how connected people feel to nature)^45–47^ and psychological restoration.^47^ Some evidence suggests that more natural environments (e.g. natural protected parks with increased biodiversity versus less biodiverse urban parks) and increased time in nature^46^ have greater effects on nature connectedness^45,47,48^ and psychological restoration.^47^ In a recent study, this relationship was more confused, with significant increases in nature connectedness seen also in urban parks as well as more natural environments.^46^ There is a clear need for more research in this area, understanding how the type, quality and dose of nature, especially in the child and adolescent population, affects health.^49^ It is important to not simply extrapolate adult findings to the child and adolescent population, as childhood and adolescence may be a particularly sensitive and unique period, whereby nature may have long-lasting effects on mental health across the life-course. For example, one European study found that adults with low levels of childhood exposure to nature had significantly worse mental health outcomes^50^ and another large study (of 1 million people) with a 28-year follow-up period, found that high levels of the presence of continuous green space during childhood was associated with a lower risk of a multitude of psychiatric disorders later in life.^51^ These findings are alarming, in light of the pattern of urbanisation and children spending less time in nature.^6^ Finally, it is important to understand what the minimal effective dose of nature may be, to inform practice and policy-making. Recommendations from a Canadian national nature prescribing programme (PaRx) suggests a minimum of 2 h in nature per week;^52^ however, it is unclear what this recommendation is based on. Coventry et al,^53^ in a systematic review and meta-analysis examining nature-based interventions in community-based adults, put forward that the most effective nature-based interventions were offered for between 8 and 12 weeks, with an optimal ‘dose’ ranging from 20 to 90 min. Current nature prescriptions take place in diverse settings,^54^ and it would be helpful to know which settings or type of nature is most beneficial.

Confounders will differ along the continuum of the nature research framework, and air pollution becomes particularly salient in human-designed environments with natural elements (e.g. urban parks) and less so in purely natural settings. Some of these confounders are seen as mechanisms driving nature's beneficial effect.^14,21^ Finally, using the biophilia hypothesis, which draws on our evolutional bond with nature, one may postulate that we may expect greater benefits of nature further up the nature research framework towards purely natural environments.

### The nature intervention

The nature ‘intervention’ falls into three key areas: access, exposure, and engagement with nature, with quantity and quality of nature being relevant to all three areas. We call this the nature intervention framework. Six out of eighteen reviews and three out of five cohort studies aimed to examine what they defined as ‘exposure’ to nature. However, five of these six reviews included studies that were not directly measuring exposure, but rather using measures of surrounding green space as a proxy,^12,26,33,37^ with the Normalized Difference Vegetation Index (NDVI) being one of the most common metrics used (Supplementary Table 1). Perhaps this is not surprising, as surrounding green space measured using the NDVI has been put forward as the most standardised metric of nature exposure.^55^ NDVI measures vegetation density.^56^ An argument against this method is that it may not accurately capture someone's exposure, as the NDVI also includes measurements of non-accessible agricultural land. Some measures of surrounding nature, such as ‘school greenness’, may be more accurate measures of true exposure, if children play outside in their school breaks. Fewer studies measure direct exposure to nature, relying on subjective reported time in green space^25,28^ or on objective measures such as real-time Global Positioning System (GPS) technology to provide participants’ daily locations and movement patterns.^12,25^ One study^57^ used geographic ecological momentary assessment (GEMA) technology (providing an objective measure of green space exposure) to assess whether being in urban green space is associated with reduced momentary psychological stress. Few reviews^27,33^ looked at exposure to nature in terms of views of nature or having contact/touching materials from nature. Some reviews used the word ‘exposure’ loosely (Supplementary Table 2) and included studies measuring surrounding nature as well as access to nature in the neighbourhood.^26,28,37^ Studies that looked at access to nature from the home or school did this in a variety of ways (Supplementary Table 1), for example measuring the time taken or distance from home to the nearest park or ‘park density’ in buffer areas around the home. Studies that examined a form of nature engagement (i.e. not merely being exposed to it but rather engaging or using it in some way) included, for example, studies looking at ‘nature play’,^29^ ‘nature-specific learning outside the classroom’,^34^ ‘outdoor education programmes’,^32^ ‘greening interventions’^41^ and the Scandinavian tradition *friluftsliv* (an immersive nature experience with an emphasis on the experience of closeness to nature during activities).^35^ Very few studies reported the quantity and quality of nature.^13^ Ensuring future studies clearly report on the quantity and quality of nature will also help us to understand the ‘dose’ required to reap its beneficial effects and what aspects of nature may be particularly beneficial or important to people's experience.

### Outcome measures

Mental health and well-being outcomes appear to fit along a continuum, with ‘disorder’ at one end and ‘well-being’ at the other. Examining outcome measures in this way has highlighted the need for a greater focus on examining nature's effects on mental disorders, including depression and anxiety. Future studies should also focus on unexamined disorders, including eating disorders, ASD, bipolar affective disorder and psychosis in children and adolescents.

This meta-review has highlighted how the current literature has often merged mental health and well-being outcomes, not fully recognising that they are related but distinct entities with a separate literature base. Zhang et al^13^ defined mental well-being as positive mental health and outcomes could include mood, stress, anxiety, depression, happiness, pleasure, emotional health, psychological health and mental health. In Fleckney & Bentley's review,^25^ even though mental health and well-being outcomes were recognised as two different entities, they found that some studies used scales/tools to measure both, thereby blurring this distinction. Bloemsma et al^39^ used a validated tool to measure mental well-being – the Mental Health Inventory-5. Future research should better recognise the distinction between mental health and mental well-being, acknowledging them conceptually and operationally as different entities, as our understanding of them has evolved.^58,59^

### Nature's effect

The evidence base is large, with the majority of studies finding significant positive results in support of nature's beneficial effect or positive results with unknown significance. Some studies found null results (not meeting significance); however, these have generally all been associated with a direction in support of a beneficial effect of nature. Only a small number of studies/findings suggested a potential negative effect, although the significance of these results is not clear. Despite the evidence base being large (in number of studies), it comes almost exclusively from HICs and there is medium to high risk of bias in the majority of studies, with few considering all potentially important confounders, such as air and noise pollution, social engagement and exercise. The mediating and moderating role of sociodemographic factors, such as ethnicity and SES, are not known or are poorly understood.

### Implications

Despite the evidence base having its limitations, we are already seeing the widespread uptake of ‘green social prescribing’ within the UK's National Health Service (NHS) as part of the government's Green Social Prescribing Programme (launched April 2021).^60^ Green social prescribing has been defined as ‘the practice of supporting people to engage in nature-based interventions and activities to improve their mental and physical health’.^60^ This could involve a health professional prescribing a community garden project to a patient with the aim of improving their mental health.^60^ Green social prescribing could therefore be thought of as a mechanism by which to implement the evidence base (on nature's effects on mental health and well-being) in practice. Despite the evidence base being larger for adults, with an attempt by Coventry et al^53^ at ascertaining the optimal ‘dose’ of nature one needs for nature-based interventions in adults, we must be mindful of the evidence base if or when we implement green prescribing to children and adolescents, understanding that even though current evidence suggests nature's beneficial effects, certainty is lacking. This is especially true for clinical populations. Intuitively, we may feel nature is good for us; however, it could be harmful (given the lack of and uncertainty in the evidence) to promote green prescribing to child and adolescent clinical populations as a form of ‘treatment’, especially if it is promoted as a stand-alone treatment and not as an adjunct to ‘treatment as usual’. However, if further down the line, more certain evidence does support its benefits, then ‘green prescribing’ could work not only for us humans (e.g. reducing the need for possible medications and unwanted side-effects) but also for the planet, in promoting nature's flourishing in our neighbourhoods and reducing the burden on mental health services (with associated carbon costs).

### Limitations

The research field examining nature and its effects on mental health and well-being in children and adolescents is a rapidly developing, interdisciplinary and heterogeneous field, mostly made up of observational studies looking at surrounding green space as a proxy for exposure in general child and adolescent population samples or non-randomised poorly controlled experimental studies in more unwell/clinical child and adolescent populations. There are few studies examining mental disorders and no studies examining disorders such as ASD, eating disorders, bipolar affective disorder or psychosis in children and adolescents. Data are lacking examining the role of ethnicity and SES in children and aolescents; however, current evidence suggests that nature may be most beneficial to those from lower socioeconomic groups or marginalised groups.

Unfortunately, not all reviews gave details of their included studies (despite our attempts to contact the corresponding authors) and therefore we were unable to identify whether reviews had missed key primary studies. However, we felt this unlikely, owing to the large number of reviews. Therefore, we did not include primary studies published before the last identified review, to avoid the risk of double counting. We limited our search to the English language, which resulted in two study exclusions. We purposefully kept the scope of this meta-review broad in order to answer our study aims; however, this made summarising the heterogeneous evidence more challenging.

It was not in our scope to formally review and report mechanistic pathways, although this could have proved useful in supporting the evidence base more formally. Data were collected on this as detailed in the protocol. Studies often framed their results in the context of current mechanistic thinking; however, few studies examined mechanistic pathways in depth or contextualised them in the context of their specific results.

### Strengths

This meta-review has synthesised a large multidisciplinary literature base that uses varying concepts and definitions and has produced two clear frameworks to guide future rigorous interdisciplinary research. Definitions and concepts of nature were broad to allow for the development of these frameworks, to ensure their relevance to this diverse field of research. This is a clear strength, as previous reviews have had a narrower focus of enquiry, which would inhibit the development of such frameworks relevant to the field at large. Studies were rigorously examined for their certainty of evidence, using validated assessment tools. A protocol was developed and made publicly available *a priori*.

### Recommendations

To date, it is difficult to judge the strength of the evidence for nature interventions for mental health and well-being in children and adolescents. Our proposed nature research and nature intervention frameworks promote the design of future studies that will bring more certainty to the evidence base, by encouraging a clearer and more consistent approach to scientifically examining nature interventions. Accumulation of more certain evidence relies on clarity and consistency of measurement, as well as the use of validated measurement tools. These factors, in turn, enable replication, which informs evaluation of the strength of evidence. If we take ‘green prescribing’ through the NHS to be a formal reflection of an evidence-based clinical intervention, then it is not acceptable or ethical for prescribing to be conducted in the absence of standard evidence to inform dosing. ‘Dose’ requires knowledge of: intervention type, how much of it and for how long, with accompanying information about expected outcomes and side-effects. In this sense, we also need to be mindful of the ‘indication’ – appreciating the difference in examining mental health and mental well-being, which are separate but connected entities, and the difference in prescribing for clinical and non-clinical child and adolescent populations, in light of the evidence base. It is also important to be aware of mediating and moderating factors that can affect intervention outcomes, including sociodemographic factors such as access to green space and sociopolitical factors such as air pollution exposure.

To increase the amount of evidence about underserved groups in mental health research, future clinical trials should focus on more ethnically diverse child and adolescent populations and children and adolescents from low- and middle-income countries. We should also expand to a wider range of diagnoses. Of the clinical child and adolescent populations, current limited research has focused on ADHD, anxiety and depression, with no research focusing on children and adolescents with ASD, eating disorders, bipolar affective disorder or psychosis.

### Conclusion

Nature appears to have a beneficial effect on the mental health and well-being of children and adolescents. However, we cannot be certain of its benefit, owing to the limitations of the evidence base coming from available studies. Even in more recent studies, not all potentially important confounders are considered (such as pollution, exercise and social engagement). To reach a global potential, more data are needed about ethnically diverse populations and populations from low- and middle-income countries.

## Supporting information

Lomax et al. supplementary material Lomax et al. supplementary material

## Data Availability

Data availability is not applicable to this article as no new data were created or analysed in this study.
