# Supplementary material for: Effect of nature on the mental health and well-being of children and adolescents: meta-review
Source: Br J Psychiatry. 2024 Sep;225(3):401–9. doi: 10.1192/bjp.2024.109 (PMC11536187; doi:10.1192/bjp.2024.109)
Supplement: Lomax et al. supplementary material [file S0007125024001090sup001.docx]

**Impact of nature on mental health and wellbeing of children and adolescents: a meta-review: Supplementary tables and figures**

Contents:

- Fig.1 PRISMA Flowchart
- Search strategies
- Table 1: Study characteristics
- Table 2: Summary Results
- List of potentially eligible studies not included in the meta-review
- Risk of bias assessments
- Fig 2. Graph showing number of nature studies published over time
- Fig 3. Graph showing number of nature systematic reviews published over time

**Fig.1: Prisma flowchart.**

**Studies included in Meta-review**

**(n= 23)** (based on levels of evidence guide)

N=18 Systematic/scoping reviews

N= 5 Cohort studies (rated good quality)

**Identification**

**Records identified from electronic databases (n= 2185)**

Medline = 805

Embase = 696

PsychInfo = 684

**Records removed *before screening*:**

Duplicate records removed

(n= 528)

**Fig 1. PRISMA flowchart: Identification of studies via databases**

**Screening**

**Records screened (Title & abstract)**

Medline, PsychInfo, Embase (n = 1657)

**Records excluded (title and abstract screening)**

Total n= 1325

**Reports sought for full text retrieval**

Total n= 332

**Reports not retrieved**

Abstracts only (n=3)

**Reports assessed for eligibility**

**(full text review)**

Total N= 329

**Reports excluded: 167**

Adult population (n = 92)

Population age not known (n=12)

Incorrect intervention (not ‘nature’) (n =41)

Non-English language (n= 2)

Protocol only (no results) (n=3)

Dissertation/thesis (n=6)

Wrong study design (n=11)

**Included**

**Eligible studies for review**

Total n= 162

**Studies not included in the meta-review (n= 139)**

Cross-sectional (n= 39)

Cohort (n= 20)

Experimental non-randomized, controlled (n=9)

Experimental non-randomized, no control (n=24)

Experimental non-randomized cross-over trial (n=4)

Qualitative study (n=10)

Narrative or rapid review (n=13)

Expert advise (n=1)

Case study/series (n=3)

RCT (n=2)

Incorrect outcome (n = 14)

**Search strategies**

Medline search strategy

1. exp adolescent/ or exp child/ or exp infant/
2. (adolescen* or babies or baby or boy* or child* or girl* or infan* or juvenil* or kid or kids or kindergarten* or minor* or neonat* or neo-nat* or newborn* or new-born* or paediatric* or peadiatric* or pediatric* or perinat* or preschool* or pre-school* or puber* or pubescen* or school * or stepchild* or teen* or toddler* or underage* or young or youngster* or youth*).ti,ab,kw.
3. 1 or 2
4. natural environment.mp.
5. Nature/
6. (mental health or mental wellbeing or cognitive development or depression or anxiety).mp.
7. forests/ or grassland/ or wetlands/ or wilderness/
8. gardens/ or parks, recreational/
9. natural world.mp. [mp=title, abstract, original title, name of substance word, subject heading word, floating sub-heading word, keyword heading word, organism supplementary concept word, protocol supplementary concept word, rare disease supplementary concept word, unique identifier, synonyms]
10. green space.mp. [mp=title, abstract, original title, name of substance word, subject heading word, floating sub-heading word, keyword heading word, organism supplementary concept word, protocol supplementary concept word, rare disease supplementary concept word, unique identifier, synonyms]
11. outdoor*.mp. [mp=title, abstract, original title, name of substance word, subject heading word, floating sub-heading word, keyword heading word, organism supplementary concept word, protocol supplementary concept word, rare disease supplementary concept word, unique identifier, synonyms]
12. forest.mp.
13. Gardening/
14. nature-based intervention*.mp.
15. ecotherap*.mp. [mp=title, abstract, original title, name of substance word, subject heading word, floating sub-heading word, keyword heading word, organism supplementary concept word, protocol supplementary concept word, rare disease supplementary concept word, unique identifier, synonyms]
16. (natur* adj3 school).mp. [mp=title, abstract, original title, name of substance word, subject heading word, floating sub-heading word, keyword heading word, organism supplementary concept word, protocol supplementary concept word, rare disease supplementary concept word, unique identifier, synonyms]
17. nature-based.mp. [mp=title, abstract, original title, name of substance word, subject heading word, floating sub-heading word, keyword heading word, organism supplementary concept word, protocol supplementary concept word, rare disease supplementary concept word, unique identifier, synonyms]
18. 4 or 5 or 7 or 8 or 9 or 10 or 11 or 12 or 13 or 14 or 15 or 16 or 17
19. (mental adj3 well-being).mp. [mp=title, abstract, original title, name of substance word, subject heading word, floating sub-heading word, keyword heading word, organism supplementary concept word, protocol supplementary concept word, rare disease supplementary concept word, unique identifier, synonyms]
20. (mental adj3 stress).mp. [mp=title, abstract, original title, name of substance word, subject heading word, floating sub-heading word, keyword heading word, organism supplementary concept word, protocol supplementary concept word, rare disease supplementary concept word, unique identifier, synonyms]
21. psychiatry and psychology (non mesh)/ or "behavior and behavior mechanisms"/ or adaptation, psychological/ or behavior/ or emotions/ or mental competency/ or motivation/ or psychology, social/ or psychosocial functioning/ or temperance/ or psychological phenomena/ or mental health/ or posttraumatic growth, psychological/ or mental disorders/ or anxiety disorders/ or "bipolar and related disorders"/ or "disruptive, impulse control, and conduct disorders"/ or dissociative disorders/ or elimination disorders/ or "feeding and eating disorders"/ or mood disorders/ or motor disorders/ or neurocognitive disorders/ or neurodevelopmental disorders/ or neurotic disorders/ or personality disorders/ or "schizophrenia spectrum and other psychotic disorders"/ or somatoform disorders/ or substance-related disorders/ or "trauma and stressor related disorders"/ or "behavioral disciplines and activities"/ or behavior control/
22. 6 or 19 or 20 or 21
23. 3 and 18 and 22
24. limit 23 to yr="2010 -Current"

Embase search strategy

1. child psychology/ or school child/ or child behavior/ or child development/ or preschool child/ or child/ or child psychiatry/ or child welfare/
2. forest/
3. recreational park/ or national park/
4. *mental disease/di, dm, pc, th [Diagnosis, Disease Management, Prevention, Therapy]
5. *mental health/
6. *psychological well-being/ or *mental health/ or *wellbeing/
7. (natur* adj3 school).ti,ab,kw.
8. (mental health or mental wellbeing or cognitive development or depression or anxiety).ti,ab,kw.
9. (mental adj3 well-being).ti,ab,kw.
10. (mental adj3 stress).ti,ab,kw.
11. 4 or 5 or 6 or 8 or 9 or 10
12. (adolescen* or babies or baby or boy* or child* or girl* or infan* or juvenil* or kid or kids or kindergarten* or minor* or neonat* or neo-nat* or newborn* or new-born* or paediatric* or peadiatric* or pediatric* or perinat* or preschool* or pre-school* or puber* or pubescen* or school* or stepchild* or teen* or toddler* or underage* or young or youngster* or youth*).ti,ab,kw.
13. *gardening/
14. *adolescent/ or *juvenile/
15. *child/ or *juvenile/ or *adopted child/ or *boy/ or *foster child/ or *girl/ or *preschool child/ or *school child/ or *toddler/
16. *infant/
17. 1 or 12 or 14 or 15 or 16
18. (natural world or natural environment or green space or eco-therap* or ecotherap* or blue space or outdoor* or park or parks).ti,ab,kw.
19. 2 or 3 or 7 or 13 or 18
20. 1 or 12 or 14 or 15 or 16
21. 4 or 5 or 6 or 8 or 9 or 10
22. 19 and 20 and 21
23. limit 22 to yr="2010 -Current"

PsychINFO search strategy

1. (adolescen* or babies or baby or boy* or child* or girl* or infan* or juvenil* or kid or kids or kindergarten* or minor* or neonat* or neo-nat* or newborn* or new-born* or paediatric* or peadiatric* or pediatric* or perinat* or preschool* or pre-school* or puber* or pubescen* or school* or stepchild* or teen* or toddler* or underage* or young or youngster* or youth*).mp. [mp=title, abstract, heading word, table of contents, key concepts, original title, tests & measures, mesh word]
2. exp Adolescent Psychiatry/ or exp Adolescent Behavior/ or exp Adolescent Psychotherapy/ or exp Adolescent Development/ or exp Adolescent Psychology/ or exp Adolescent Psychopathology/ or exp Adolescent Health/
3. exp Child Psychotherapy/ or exp Child Welfare/ or exp Child Psychopathology/ or exp Child Health/ or exp Child Psychiatry/ or exp Child Behavior/ or exp Child Psychology/
4. exp Infant Development/ or exp Infant Temperament/
5. 1 or 2 or 3 or 4
6. (natural environment or natural world or green space or blue space or outdoor* or nature-based intervention* or eco-therapy* or ecotherap* or nature-based).mp. [mp=title, abstract, heading word, table of contents, key concepts, original title, tests & measures, mesh word]
7. (nature adj3 school).mp. [mp=title, abstract, heading word, table of contents, key concepts, original title, tests & measures, mesh word]
8. exp "nature (environment)"/
9. exp Horticulture Therapy/ or gardening.mp.
10. exp Wilderness Experience/
11. (park or parks).mp. [mp=title, abstract, heading word, table of contents, key concepts, original title, tests & measures, mesh word]
12. 6 or 7 or 8 or 9 or 10 or 11
13. (mental health or mental wellbeing or cognitive development or depression or anxiety).mp. [mp=title, abstract, heading word, table of contents, key concepts, original title, tests & measures, mesh word]
14. (mental adj3 well-being).mp. [mp=title, abstract, heading word, table of contents, key concepts, original title, tests & measures, mesh word]
15. (mental adj3 stress).mp. [mp=title, abstract, heading word, table of contents, key concepts, original title, tests & measures, mesh word]
16. *Mental Health/
17. *Mental Disorders/
18. 13 or 14 or 15 or 16 or 17
19. 5 and 12 and 18
20. limit 19 to yr="2010 -Current"

**Table 1: Study characteristics of included studies in the meta-review**

| **Table 1: Study characteristics of the 16 systematic and 2 scoping reviews and 5 cohort studies included in the meta-review.** | | | | | |
| --- | --- | --- | --- | --- | --- |
| ***Systematic and scoping reviews*** | | | | | |
| **Author** | **Population**  **Setting** | **Intervention or exposure**  **Comparator** | **Outcome**  **Follow up** | **Study design**  **Total no. of included studies in the review** | **Key Results**  **Ethnicity & socio-economic status (SES)** |
| **Alderton 2019** | Population:  Children < 8 years.  General population-based samples (‘non-representative  samples’ e.g.: in-patient clinical samples were excluded).  Setting:  Urban setting.  High- income countries (HICs) located in North America (USA) & Europe (UK, Germany, Spain, Netherlands). | Intervention or exposure:  Quality, quantity and/access to neighborhood public open space: e.g.: some studies looked at distance to nearest park, some access to private garden and some residential greenness in buffer areas around home.  (Note: review also examined literature on housing quality, density, typology/affordability, and quality and/access to local social health municipal for early childhood education and care services)  Comparator:  No limits made on comparator | Outcome:  Any measure of competence (e.g.: social competence, emotional maturity) or difficulties or disorders (e.g.: conduct problems, hyperactivity or ADHD).  Follow up:  Not pre-defined or clearly reported in all studies. One cohort study had a 1-2 year follow up. | Study design  Systematic review of peer-reviewed quantitative studies.  No reported protocol.  1 reviewer searched ProQuest Central, PubMed and Google Scholar up to 17th Jan 2019.  No formal quality assessment tool used to assess included studies. Performed a descriptive analysis.  Total no. of included studies  14 | Studies tended to report associations with better mental health. All 5 studies examining mental health difficulties found at least one significant difference in reducing difficulties. 2 found a borderline significant association in the opposite direction, emerging when only looking at higher education groups & ADHD. 2 out of 3 studies examining mental health competence found a significant association in improving competence, with only one finding an association in the opposite direction with borderline significance.  9 of 14 studies included some measure of SES, however only 3 compared findings across socio-economic groups. 2 of these 3 studies found benefits to be greater in those from poorer socioeconomic backgrounds. Only 5 of the 14 studies collected data on ethnicity. |
| **Bikomeye 2021** | Population:  Children <18 years. No pre-specification over general or clinical population.    Setting:  School/daycare yard. Mostly urban settings (one study in a rural setting). Studies are from HICs (however some studies focus on deprived areas within these countries): Netherlands, Austria, USA, UK (2 studies), Canada. | Intervention or exposure:  Experimental school greening interventions. 4 studies used ‘traditional schoolyard greening interventions’ (outdoors changed to include natural elements e.g., trees, flowers), 2 used ‘modified versions of such interventions’, 1 involved adding an ‘AstroTurf’ & 1 focused on “Social and Therapeutic Horticulture (STH)” (emphasizes active engagement of children in creation of green spaces).  Comparator:  Main comparator: pre to post intervention, however also included studies with a school non-greening intervention control arm. | Outcome:  Physical health or socioemotional health (SEH). SEH measured in a variety of ways and not pre-defined by the review.  Follow up:  Varied between studies and not pre-defined by the review. | Study design:  Systematic review of experimental studies.  No reported protocol.  2 reviewers searched 4 databases: Ovid, Medline, PsycINFO, Scopus & Greenfile up to 20 July 2020.  No formal quality assessment tool used to assess included studies. Performed a descriptive analysis.  Total no. of included studies  6 | All 6 studies reported beneficial effects on socioemotional health in the majority of outcome measures, however some found no effect on certain measures (van Dijk-Wesselius found no effect on emotional well-being, perceived restorative quality & older children’s prosocial behaviours but did find a positive effect on attention restoration). 2 results showed negative associations (non-significant or significance was not reported), in measure of prosocial behaviour (Brussoni et al.) and in well-being outcomes (Chiumento et al). This conflicted with their other results (e.g.: Chiumento et al. found a positive impact on socioemotional health in qualitative data).  Only 2 studies adjusted for some measure of SES and only one study adjusted for ethnicity. |
| **Dankiw 2020** | Population:  Children aged 2-12 years with no pre-existing/diagnosed physical, mental, behavioral or neurological health condition.  Setting:  Not pre-defined, however all studies involved a ‘nature play space’. Studies were conducted in largely HICs: Norway (n= 3 studies), Slovenia (n=1), Australia (n=2), United Kingdom (n=1), Scotland (n=1), Canada, (n=1), United States of America (n=4) and Germany (n=4). | Intervention or exposure:  Unstructured free play within nature (called ‘nature play’), including natural elements.  Comparator:  Not pre-defined, however largely used traditional play space vs nature play space as the comparator. | Outcome:  Any outcome related to nature play-including gross motor, social, cognitive development, learning, emotional or mental health and physical health.  Follow-up:  Not pre-defined or clearly reported in all studies. | Study design:  Systematic Review of quantitative studies. Protocol available on PROSPERO. 4 reviewers searched 7 databases: MEDLINE, ERIC, Embase, PsycINFO, The Cochrane Library, The Joanna Briggs Institute and Emcare up to July/Aug 2019. Searched grey literature. The McMaster Critical Appraisal Tool (CAT) for Quantitative Studies was used to assess quality of included studies.  Performed a descriptive analysis.  Total no. of included studies:  16 | 5 studies examining cognitive development consistently reported positive improvements (however significance not always reported). Cognitive development was examined by looking at forms of play (functional, constructive, explorative, dramatic & imaginative), learning & creativity. 3 of 4 studies examining social outcomes found an improvement & 1 found no difference (between traditional & nature play). 2 studies examined emotional outcomes- 1 found a significant decrease in aggression & depression & other found a positive improvement in mood. There were concerns around sampling bias, reporting, reliability & validity of outcome measures. Studies often lacked a suitable control.  Dankiw commented that most studies were done in Western countries with majority Caucasian populations, but that most studies did not detail sample characteristics, specifically ethnicity. They did not report or discuss SES. |
| **Fang 2021** | Population:  Adolescents age 10-19. Studies included at risk/unwell populations as well as more general populations.  Setting:  Varied depending on location of OEP. Some populations recruited from schools, some based on socio-economic or at-risk factors e.g.: low-income households. All from HICs: USA (N=5), Israel, Australia (n=2), Canada, Germany, New Zealand (n=2), Hong Kong, UK, Norway. | Intervention or exposure:  Outdoor Education Programs (OEPs) defined as 'an intervention that included outdoor-related activities e.g.: outdoor camping, wilderness adventure, nature-based field trips, resident camp, backpacking trip.’  Comparator:  Studies selected had to have comparative groups (i.e.: experimental and control group comparisons), however results include some studies with pre- and post- OEP outcome measures. | Outcome:  Any outcome measure of ‘self-efficacy’. Examples of self-efficacy measurement tools include Self-Efficacy scale or the Generalized Self-Efficacy scale.  Follow-up:  Follow-up time not pre-defined, however detailed follow-up clearly in results table and varied from no follow-up to 24-month follow-up. | Study design:  Systematic review or observational & randomized studies. No reported protocol.  2 reviewers searched 6 databases: PubMed, ScienceDirect, Medline, PsycArticles, Behavioral sciences Collection of EBSCO and Eric from Jan 2000 to 31^st^ Oct 2020.  Used the tool MINORS (Methodological Index for Non-  randomized Studies) to assess study quality.  Performed a meta-analysis.  Total no. of included studies:  12 | In their meta-analysis they found that adolescents participating in OEPS experience improvements in their self-efficacy, but this was moderated by their mental health, length of the OEP and study groups. There was significant heterogeneity but no evidence of publication bias.  Neither ethnicity or SES were reported or discussed in the review |
| **Fleckney 2021** | Population:  Adolescents 10–19 years. Excluded studies that exclusively focused on narrowly-defined subgroups (e.g. youth with autism) or institutional settings (e.g. school grounds, hospitals),  16/24 studies drew samples from nationally representative population-based surveys.  Setting:  Focused on ‘urban public realm’ outside home & school. 12 papers included urban & rural settings & 12 focused on urban populations. Mostly from HICs: USA (n=10); UK (n=4); Europe (n=3), Canada (n=2); New Zealand (n=2), Australia (n=1). Only 2 studies from global South (Iran; Caribbean countries). | Intervention or exposure:  Examined more broadly the ‘urban public realm’, however a key focus was on urban green space with 19 of 24 studies examining exposure to proximate green space. Measures of green space exposure varied with some measuring a proxy of exposure e.g.: local ‘park density’ whilst others measured ‘self-reported use’.  Comparator:  Not pre-defined/specified. | Outcome:  Outcomes relating to one/more of following: depression, anxiety, stress or mental, emotional, psychological wellbeing.  20/24 studies measured mental health outcomes in regards to disorder or difficulties such as anxiety, depression or stress. 8 looked at psychological, emotional or mental wellbeing outcomes. 23/24 relied on self-report data. 2 studies supplemented self-report data with parent or teacher report data.  Follow-up:  Not pre-defined. Reported in some longitudinal studies in descriptive analysis (1 had a 3 year follow up). | Study design:  Systematic review of original empirical research. No reported protocol. 1 reviewer performed a literature search in PsychINFO & Scopus from 1^st^ January 2005 to 27^th^ March 2020. No formal quality assessment tool used.  Performed a descriptive analysis.  Total no. of included studies:  24 studies, with 19 focusing on proximate greenspace. | Of the 15 cross-sectional studies examining green space, 11 found greenspace to be beneficial for adolescent mental health or well-being (9 having statistically beneficially effects). The evidence from longitudinal studies were less clear, as only 4 studies were identified and all had moderate to serious risk of bias and had mixed results.  13 studies adjusted for ethnicity. 10 studies adjusted for household SES or income and 10 adjusted for neighbourhood deprivation. |
| **Fyfe-Johnson 2021** | Population:  Under 19 years. No specification re sample type (general population vs clinical/unwell population).  Setting:  Not pre-specified, however included studies on residential green space (n= 147); school greenspace (n=40) and ‘General Green Space Activity’ (n = 35). No information on country of origin of included studies. Reported  few studies examined marginalized communities. | Intervention or exposure:  Examined nature exposure which they defined as: gardening, green space activity (e.g.: green space not near residence or school), residential green space, school green space, greening intervention, wilderness experience, nature walk, and 'other' (studies where mixed spaces were examined).  Comparator:  Not pre-defined/specified. | Outcome:  Collected data on 7 different categories of outcomes: cognitive, behavioral & mental health outcomes; academic & learning outcomes; physical activity; BMI; CV & metabolic measures; asthma and allergy and ‘other’. Mental health outcomes included a range of outcomes from psychological wellbeing to mental health diagnoses. Academic & learning outcomes were limited to those most related to classroom performance.  Follow-up:  Not pre-defined or reported. | Systematic review of original studies. Was registered in  the International Prospective  Register of Systematic Reviews database (Registration number:  CRD42018087552).  2 reviewers searched PubMed, Cumulative Index to Nursing and Allied Health Literature, PsycInfo, ERIC, Scopus & Web of  Science databases, up to 23^rd^ Feb 2021. Quality assessment tool used to assess included studies: Mixed Methods Appraisal Tool (MMAT). Performed a descriptive analysis.  Total no. of included studies.  296 | Of the 85 studies looking at cognitive, behavioural and/mental health outcomes, 71 studies (83%) found positive results, 12 studies (15%) reported mixed/null results and only 2 (2%) reported negative associations. There were 5 RCTs identified and all 5 reported positive associations with well-being, mental fatigue & recovery, cognitive tasks, emotional status, performance tasks and perceived restoratives and stress. They concluded overall evidence suggests nature’s beneficial role, however more high-quality evidence is needed and further evidence examining marginalized communities who tend to be most at risk of poor access to green space.  Did not report on ethnicity or SES of included studies, however commented that few studies examined marginalized communities who may be most at risk of inequitable access to green space. |
| **de Keijzer 2016** | Population:  Included studies of all ages, with a subgroup analysis on children/adolescents. Of the 6 studies included on children/adolescents, there was a mix of general school populations, at-risk and clinical populations.  Setting:  3 studies focused on public high/elementary school children, 2 on children with ADHD (ages 7-12 and 5-18 respectively), and one focused on low-income urban families. All studies were from HICs: 5 from USA and 1 from Spain. | Intervention or exposure:  Inclusion criteria: At least one of the exposures was a measure of green space/natural environment, including outside activities in green spaces such as gardening. Of the studies included in the child/adolescent age group, 3 studies looked at surrounding greenness to school or home, one looked at views of greenness at school and 2 looked at activities in a 'green setting'.  Comparator:  Not pre-defined/specified. | Outcome:  At least one outcome measure of cognition, attention, dementia, or Alzheimer’s, (outcomes such as stress, relaxation, mood & mental health were excluded). Attention/inattention was the most studied outcome measure in the child/adolescent age group.  Follow-up:  Not pre-defined or clearly reported in all longitudinal studies. | Study design:  Systematic review of observational studies. No reported protocol.  Number of reviewers for article screening unclear. Searched MEDLINE & Scopus (Web of Science) up to 26^th^ May 2016.  Quality assessment tool used was one that had been developed from a previous review looking at green space on mental health (Gascon et al. 2015). Performed a descriptive analysis.  Total no. of included studies:  13, with 6 on the child/adolescent age group. | 4 out of 6 studies that looked at school performance, cognitive development/attention found a beneficial association. 1 large longitudinal study (n= 2623) found greater progress in working memory & reduced inattentiveness. 2 ecological studies found a positive association (between views of nature & school level scores & graduation rates and between school surrounding greenness and English & Maths scores). 1 cohort study found an improvement in children’s attention after moving to a house with more surrounding greenness. 2 studies found a positive association of green exposure & symptom improvement in ADHD. Concluded green space appears good for cognition, despite concerns over inadequate controlling of confounders & no studies looking at green space quality.  De Keijzer found that all studies except one adjusted for a measure of socioeconomic status, however no study investigated effect modification of socioeconomic status (e.g.: comparing across socioeconomic strata). They did not report or discuss ethnicity in their review. They called for future studies to investigate the effect modification of SES and ethnicity. |
| **Luque-García**  **2022** | Population:  Children between ages 6-12 years; aim to obtain studies representative of primary school children. (Excluded pre-school children, teenagers and adults). Most from school or general population samples/cohorts (i.e.: not unwell/clinical populations).  Setting:  Mix of studies looking at residential, neighborhood & school surrounding green space. Of 34 included studies, 17 were from Europe, 9 from USA, 4 from Australia, 2 from South Korea, 1 from Canada & 1 from China. | Intervention or exposure:  Studies were included if they examined exposure to landscapes dominated by vegetation assessed by objective (e.g.: vegetation indices, land cover data) or subjective methods (e.g.: questionnaires). Greenness exposure was measured differently across studies, most using surrounding greenness around the home, school/neighborhood. Most widely used (n=15) objective measure of greenness was the NDVI.  Comparator:  Pre-specified as comparatively lower levels of exposure or lack of exposure. | Outcome:  Neuropsychological development & mental health outcomes assessed via validated tools. Excluding studies that examined outcomes in terms of medical diagnoses. Neuropsychological & mental health outcomes were diverse in the studies & included: attention, working memory, intelligence, cognitive development, academic performance, well-being, ADHD symptoms and behavior.    Follow-up:  Not pre-defined or reported for all follow-up studies (e.g.: cohort studies) | Study design:  Systematic review of observational studies. Protocol registered on PROSPERO. 3 reviewers searched PubMed, Scopus, Web of Science & EBSCO’s GreenFILE up to 5^th^ Oct 2021. Assessed risk of bias with the Navigation Guide methodology. Performed a descriptive analysis.  Total no. of included studies:  34 | They reported that exposure to greenness seems to have a predominantly beneficial effect on neuropsychological development & mental health of children, however due to the high risk of bias and heterogeneity in methodology they felt it was difficult to draw firm conclusions. Highest risk of bias related to the exposure assessment (many did not assess green space quality, accessibility, actual green space use or exposure time).  5 of the 34 studies controlled for ethnicity. All studies except 2, controlled for some measure of SES. |
| **Mann 2022** | Population:  School-aged participants (Kindergarten to Year 12) sampled from schools.  Setting:  Setting was a ‘natural outdoor setting’ including e.g.: school grounds/gardens or local green space like forests. Most studies were conducted in USA (n=54), followed by UK (n=27), Australia (n=15), Canada (n=8), Denmark (n=6), NZ (n=6), Spain (n=4), South Africa (n=4), Sweden (n=3), Germany (n=2), Singapore (n=2) and Turkey (n=2). | Intervention or exposure:  Learning activities outside in nature called ‘NSLOtC’ (nature-specific learning outside the classroom), which includes practical & experiential learning activities conducted outside in school grounds & other locations such as parks, forests, residential camps/centers or on expeditions.  Comparator:  Included studies with ‘within-subject comparisons’, ‘between-subject comparisons’ (e.g.: no treatment or treatment-as-usual) and studies without controls. | Outcome:  School student wellbeing, academic or socio-emotional outcomes. The most common reported outcomes were described as “soft skills” i.e.: relating to a student’s developing understanding of their self-concept & intrapersonal skills (e.g.: resilience & self-confidence) & their social & interpersonal skills (e.g., communication and teamwork).  Follow-up:  Not pre-defined or specified. | Study design:  Systematic review of original research studies. Protocol registered on PROSPERO (CRD42020153171).  1-2 reviewers searched 9 databases (1 reviewer screened title and full text): ERIC, ProQuest, PSYCInfo, PubMed, Sage, Scopus, Taylor and Francis, Web of Science & Wiley from 2000 to 2020. Quantitative studies were assessed using the CCEERC tool. Qualitative studies were assessed with the JBI Checklist. Performed a descriptive analysis.  Total no. of included studies:  147 | They concluded nature-specific outdoor learning has noticeable socio-emotional, academic and wellbeing benefits and felt the evidence was significant enough to recommended that NSLOtC should be part of national curricular. They reported the quality of evidence wide ranging but generally moderate. They also recommended training should be given to teachers on how to provide this as well as more research being conducted with greater rigorous designs, looking more thoroughly across age groups and learning settings.  Neither ethnicity or SES were reported or discussed in the review |
| **McCormick**  **2017** | Population:  Children aged 0-18 years. Populations mostly came from school samples (high school/elementary/public school) with only one study on a clinical population (children with ADHD).  Setting:  Studies came from rural & urban settings. Did not report on study’s country of origin. | Intervention or exposure:  ‘Greenspace’. Of the included studies, some looked at greenspace exposure- (e.g.: high-school greenness), some looked at access (time it takes from home to get to a greenspace) and some looked at views of nature from the home.  Comparator:  Not pre-defined/specified. | Outcome:  Mental health & well-being outcomes.  Outcomes included: perceived restorativeness, ADHD/ADHD symptoms, stress, competence, cognitive development, behavioral problems, school performance & 'mental health’ utilizing the strength and difficulties questionnaire.  Follow-up:  Not pre-defined or specified in table of study characteristics | Study design:  Systematic review. (Study type for inclusion not specified). No reported protocol. 1 reviewer searched 2 databases: Ovid and PubMed from 2012 to 2017. No formal quality assessment tool used. Performed a descriptive analysis.  Total no. of included studies:  12 | They concluded the beneficial association of nature, specifically access to nature and improved mental well-being, overall health and cognitive development. They noted improved attention restoration, memory, self-discipline, reduced stress, improved behaviour and symptoms of ADHD, higher standardized test scores and greater competence and development of supportive social groups.  Neither ethnicity or SES were reported or discussed in the review |
| **Mygind 2019** | Population:  Children & adolescents (up to 18 years). Participants were largely 11 -18 years (only 10% of studies included participants under 11). Participants included typically developing children/ adolescents, as well as children/ adolescents with behavioral and/or emotional disturbances (e.g. ADHD, depression).  Setting:  Any setting delivering a nature-immersive experience (studies were wide-ranging). Studies came from USA (n=14), England (n=5), Israel (n=3), Japan (n=1), Singapore (n=1), Germany (n=1), Norway (n=1), NZ (n=8), Scotland (n=1), Netherlands (n=1). | Intervention or exposure:  'Immersion in natural environments' defined as non-competitive  activities, both sedentary & active, occurring in natural environments removed from everyday environments. There is an emphasis on the experience of closeness to nature during an activity, known as ‘friluftsliv’ (Scandinavian practice).  Comparator:  Comparator group for experimental studies: urban or indoor setting. | Outcome:  Examined 'mental, physical and social health outcomes'. Mental health outcomes included: Self-esteem, self-efficacy, self-concept, resilience, problem solving, academic performance, cognitive performance, mood, psychophysiological stress, skills and behavioral indicators.  Follow-up:  Not pre-defined, however well reported. Ranged from 1 day to 9 months. | Study design:  Subsample (child/adolescent population) of a Systematic review of quantitative studies with a wider scope (all ages). Original review protocol registered on PROSPERO  (CRD42017057988). 2 reviewers searched Dissertation Abstracts, ERIC, PsycINFO, Scopus, SPORTDiscus, Web of Science up to May 2017. Quality assessment method was adapted from Cochrane Collaboration's tool for randomized trials. Performed a descriptive analysis.  Total no. of included studies:  84 studies included in narrative synthesis. 36 studies subjected to quality assessment and synthesis. | In regards to mental health outcomes, 65.7% of studies (n=23) reported greater improvements following immersive nature experiences compared to control conditions/groups, 8.6% (n=3) showed improved outcomes but no more than controls and 25.7% (n=9) had mixed or insignificant findings. The evidence appeared greatest for benefits on self-esteem, self-efficacy, resilience and academic and cognitive performance. There was some evidence in support of skill-orientated and behavioural indicators (although they were assessed in different ways between studies). Results were more inconclusive when looking at the outcomes of self-concept, problem solving and mood. The main sources of bias were in the sampling and use of unsuitable comparison groups.  Neither ethnicity or SES were reported or discussed in the review |
| **Reece 2021** | Population:  14-24 years old. Most studies (94%) were of the general population. The remainder involved clinical/self-diagnosed populations with anxiety &/ depression (6%).  Setting:  Populations were from urban settings. Most studies were carried out in North America (28.2%), followed by Europe (21.8%), UK (17.3%) and Asia (15.8%). | Intervention or exposure:  Exposure to greenspace/nature. The most commonly studied exposure was urban green space (n=41, 20%) followed by exposure/contact/touching materials from nature (n=40, 19.5%) & forest or woodland experiences (n=25, 12%).  Comparator:  Not pre-defined, however experimental studies needed to include  a comparator/control group for inclusion. | Outcome:  Anxiety & depression outcomes. Of the included studies, outcome measures included measures of anxiety (n=17; 8.5%), depression (n=11; 5.4), both anxiety & depression (n=26; 13%) & ‘other’ (e.g.: measures of mood, well-being, stress, quality of life, self-esteem) (n=147; 73.1%). Few studies used clinical measures of anxiety/depression.  Follow-up:  Not pre-defined or specified. | Study design:  Scoping review of quantitative & qualitative studies. No reported protocol. 2 reviewers searched 7 databases from Jan 2000 to June 2020: Medline, PsycINFO, Scopus, CINAHL plus, Global Health & CAB Abstracts. Grey literature was also searched using Open Grey. No critical appraisal. Performed a descriptive analysis.  Total no. of included studies:  201 | Of the studies identified, the majority (94%) were on the general population, verses populations with clinically or self-diagnosed anxiety and/depression (6%). Most studies reported on general mental health outcomes (e.g.: low mood, stress) rather than clinical measures of anxiety or depression and therefore the review concluded more evidence is needed looking at clinical measures of anxiety or depression.  Reported that 198 included studies (98.5%) did not report on ethnicity. SES was not reported or discussed in the review. |
| **Stea 2022** | Population:  Children & adolescents (6–18 years) with self-reported mental health difficulties/diagnosis & their families (at least one caregiver had to be involved in treatment). Most participants were referred due to substance abuse, behavior problems or delinquent activity, poor family relationships and or emotional dysregulation/mental illness.  Setting:  Not pre-defined, identified as intervention setting for family-based outdoor therapy. All included studies (n = 7) were from the United States. | Intervention or exposure:  Family-based therapy taking place outdoors, including for example adventure therapy, ecotherapy, outdoor rehabilitation, nature-based programs, wilderness programs, forest bathing &/ bushcraft (role of nature had to be intended to have therapeutic benefit).  All studies included individual and/ group-based counselling processes.  Comparator:  Not pre-defined. All 7 studies were experimental. 3 studies had no control/comparison group. There was no randomization to treatment vs comparison/control groups. | Outcome:  ‘Mental health problems'. Study outcomes included: family functioning, self-concept, self-perception, problem behavior/behavioral problems, adolescent attachment or unresolved attachment, parent & peer attachment, competence & problems in children, impact of trauma.  Follow-up:  Not pre-defined, however clearly reported. Ranged from 21 days to 12 months. | Scoping review of peer-reviewed primary research studies (any design). Reported to have a pre-defined protocol however not accessible. 3 reviewers searched Medline, Embase, APA, PsychInfo, AMED, Scopus, CENTRAL trials database  (Cochrane library) & CINAHL with no restriction on year (no date hence assume year of publication - 2022). No critical appraisal/quality assessment of included studies. Performed a descriptive content analysis.  Total no. of included studies:  7 | They concluded that family based outdoor treatment positively impacts family and peer relationships, adolescent behaviour, mental health, self-perceptions/self-concept, social engagement, delinquency rates and school success. However most studies included few participants and lacked comparable control groups, meaning that it was difficult for the authors to draw firm conclusions.  Reported that most studies included families of Caucasian ethnicity. Reported there was a lack of socioeconomic diversity in the included studies. |
| **Sprague 2022** | Population:  2- 18 years. Most studies appear to be observational studies from general population samples, however population type (clinical vs non-clinical not pre-specified).  Setting:  Not predefined, however most studies (75%) focused on residential greenspace. Most studies came from wealthy, Western European countries,  No studies from LMIC (low & middle income countries) | Intervention or exposure:  Examined green space exposure. Studies looked at green space exposure in 4 ways: distance to greenspace, type of greenspace (e.g., parks, gardens), use of greenspace (e.g., walking, playing) and frequency of exposure. Most studies (n = 21, 75%) focused on residential distance to greenspace.  Comparator:  Not pre-defined/specified. | Outcome:  Developmental outcomes, measuring outcomes related to physical health, cognitive functioning, socioemotional wellbeing, and mental health. Of the 28 included studies, outcomes fell into the following groups: cognitive & brain development (n = 6; 21.4%), mental health & wellbeing (n = 13, 46.4%), attention & behavior (n = 8, 28.6%), allergy & respiratory (n = 3, 10.7%) & obesity & weight (n = 2, 7.1%).  Follow-up:  Not pre-defined or specified. | Systematic review of longitudinal or experimental studies. No reported protocol. 2 reviewers screened Cochrane, Web of Science, PubMed, CINAHL, Scopus & Environment Complete databases up to 9^th^ October 2020. Qualitative assessment done using a 10-item quality assessment checklist adapted from a previous review (Lachowycz & Jones 2011) and inductive thematic analysis (Braun & Clarke’s six-step method). No meta-analysis.  Total no. of included studies:  28 | 6 studies were identified looking at cognitive & brain outcomes, with 3 finding positive associations (in working memory, cognitive performance & several white & grey matter regions of brain) and 3 no association. 13 studies looked at mental health & well-being & all found positive associations (moderate quality evidence). Outcomes included improved prosocial behaviour, mood, emotional resilience, self-determination, happiness, tonic vagal tone & reduced risk of developing schizophrenia & psychiatric disorders. 8 studies looked at attention & behaviour, with 7 reporting positive associations & 1 no association. Outcomes included improvement in prosocial behaviour, self-regulation, attention, reduced peer & conduct problems & decreased odds of having ADHD (moderate evidence base).  Reported that there was limited reporting of ethnicity in included studies (24/28 did not report on ethnicity). 15/24 studies adjusted for some measure of SES. |
| **Tillmann 2018** | Population:  Children/adolescents 18 years and under. Early adolescence was the most commonly studied group. Population type (general vs unwell populations) not pre-specified however there appears to be a mix of general population & clinical populations e.g.: those with ADHD.  Setting:  Not pre-specified and varied greatly from city parks to wilderness settings. Of the 35 studies, 11 were conducted  in the USA, 8 in the UK, 2 in Canada & the remaining 14 in  other countries. | Intervention or exposure:  Intervention that 'incorporated an element of nature'. There was large variation in the specific elements of nature looked at, with 6 studies including green space, 2 water/ ‘blue’ space, 4 greenness/greenery, 2 vegetation (e.g.: grass, trees), 1 gardens, 4 parks, 8 outdoor programs/education, 4 wilderness therapy, 1 forest school & 9 at various outdoor/natural settings (e.g.: schoolyards, green outdoor settings). They grouped these into 3 broad categories ‘accessibility’, ‘exposure’ or ‘engagement’ with nature.  Comparator:  Not pre-defined/specified. | Outcome:  Any outcome that includes a component of mental health. Outcomes were assigned to 1 of 8 categories: emotional well-being (n=15), attention deficit disorder/ hyperactivity disorder (ADHD) (n=10), overall mental health (n=9), self-esteem (n=9), stress (n=4), resilience (n=3), depression (n=3) & health-related quality of life (HRQOL) (n=2). Majority focused on emotional well-being & ADHD.  Follow-up:  Not pre-defined or specified. | Study design:  Systematic review of primary studies. Protocol available on PROSPERO (CRD42016046085). Searched 10 databases (unclear by who) from 1990 to 1^st^ March 2017: PubMed, Scopus, PsycINFO, Geobase, ProQuest, SPORTDiscus, Sociological Abstracts, Leisure & Tourism Database, Physical Education Index & EMBASE. Quality was assessed using quality assessment tools developed by the National Heart, Lung, and Blood Institute of the U.S. National Institutes of Health Quality Assessment Tools. Performed a descriptive analysis.  Total no. of included studies:  35 | Of the 35 studies identified, half of all findings showed statistically significant positive associations between nature and mental health outcomes, nearly half reported no statistical difference and only 1 paper reported a single finding suggesting nature had negative effects. Most significant positive findings were for outcomes relating to ADHD, overall mental health, stress, resilience & health-related quality of life. Evidence for outcomes: emotional well-being, self-esteem & depression were more inconclusive (greater number of non-significant findings than positive significant findings).  Neither ethnicity or SES were reported or discussed in the review |
| **Vanaken 2018** | Population:  Children, adolescents or young adults (age limit 25 years). Not pre-specified whether looking at general or unwell populations. Only 3 of 21 included studies examined populations that included young adults (up to 25).  Setting:  Most studies examined residential greenspace. Some looked at green space around the school. 11 studies were conducted in Europe, others were conducted in North America (n= 6) & Oceania (n= 4). | Intervention or exposure:  Looked at green space  exposure in residential/school surroundings. Exposure was measured in the majority of studies with at least one objective parameter e.g.: 10 studies used land cover maps & 9 used remote sensing data based on satellite images (Normalized Difference Vegetation Index: NDVI). These could be considered proxy measures of exposure. Only 1 study used geolocation data obtained by study participants wearing digital watches with GPS-functionality.  Comparator:  Not pre-defined/specified | Outcome:  Mental health & neurocognitive development assessed using standardized instruments/through existing medical records/ databases. Included studies fell into 3 outcome categories: emotional & behavioral difficulties, mental well-being & neurocognitive development.  Follow-up:  Not pre-specified, some reporting of follow-up in longitudinal studies in table of characteristics. | Study design:  Systematic review of observational studies. No protocol.  Searched PubMed and Scopus up to 2^nd^ March 2018 (no of reviewers not made explicit).  No formal quality assessment tool used.  Performed descriptive content analysis.  Total no. of included studies:  21 | Reviewing 21 studies gave consistent evidence in support of the beneficial association between green space exposure & children’s emotional & behavioural difficulties (especially hyperactivity & inattention problems). Evidence was more limited but still indicating a beneficial association in mental wellbeing of children or depressive symptoms in adolescents. Associations were still seen after studies adjusted for demographic & socioeconomic confounders.  Reported that there were a lack of studies adjusting for ethnicity or neighbourhood SES. |
| **Zare 2022** | Population:  Children, up to 18 years old. Total of 29 studies, with population ages that varied - with some focusing on young children and some on older children.  Excluded patients or non-healthy populations.  Setting:  Not pre-defined or reported.  Most studies conducted in Europe (n = 14; 3 in Germany, 2 in Lithuania, 2 in Spain, 2 in Belgium, 4 in UK, 1 in Denmark), followed by USA (n = 8). 3 studies were from Asia (2 in China, 1 in South Korea) & 4 studies were from Oceania (3 in Australia, 1 in New Zealand). | Intervention or exposure:  Long-term exposure to green space.  Most studies used greenspace availability indicators (surrounding greenness, percentage of land cover) as a measure of exposure. Greenspace use and quality were rarely considered.  Comparator:  Reported using the PECO (Population, Exposure, Comparator, Outcome) and the comparator as ‘not applicable’. | Outcome:  Behavioral outcomes.  From the included studies, they identified nine types of behavioral outcomes including: total behavioral difficulties (n = 16), ADHD symptoms & severity (n = 15), conduct problems (n = 10), ADHD diagnosis (n=10), prosocial behavior (n =10), emotional symptoms (n =8), peer-relationship problems (n = 8), externalizing disorders (n =6), and internalizing disorders (n = 5).  Follow-up:  Not pre-defined or clearly reported for all longitudinal studies. | Systematic review of observational quantitative studies. Reported there was a pre-defined protocol however not published/accessible.  Two teams of reviewers searched three databases: PubMed,  Scopus, and Web of Science, up to 29^th^ November 2021.  Risk of bias was assessed by the Newcastle–Ottawa Scale (NOS).  Performed a descriptive content analysis.  Total no. included studies:  29 | Reviewed 29 studies, 16 studies used the Strength and difficulties Questionnaire to assess total behavioural difficulties. Other behavioural outcome measures included ADHD symptoms & severity (n = 15), ADHD diagnosis (n = 10), conduct problems (n = 10), prosocial behaviour (n = 10), emotional symptoms (n = 8), peer-relationship problems (n = 8), externalizing disorders (n = 6) & internalizing disorders (n = 5). They reported most of the available studies to have fair/good quality and that the findings supported a potential association between greenspace exposure & reduced risk of behaviour problems.  Lack of studies adjusting for neighbourhood SES (mostly adjusted for household SES) and ethnicity. |
| **Zhang 2020** | Population:  Adolescents aged 10 - 19 years or those at high school, secondary school, junior high or intermediate school. Did not specify population type (e.g.: general or unwell/clinical population).  Setting:  Publicly accessible open green space e.g.: forests, parks, gardens. 5 studies were conducted in USA, with the remainder from the Netherlands (n= 2), UK (n= 2), Canada, Germany, Austria, Australia and New Zealand (all n= 1). | Intervention or exposure:  Studies with an objective or subjective measure of green space exposure (including quantity or quality of exposure) in the school or residential environment, (where green space was defined as urban areas of vegetation).  Only 2 studies assessed both perceived & objective measures of green space. One study assessed green space quantity and self-reported green space quality.  Comparator:  ‘Comparison/control groups were not required for articles to be included in the review’. | Outcome:  Mental well-being outcomes included any of the following: mood, stress, anxiety, depression,  happiness, pleasure, emotional health, psychological health, and mental health.  Follow-up:  Not pre-defined or clearly reported for all applicable studies. | Systematic Review of cross-sectional, longitudinal, experimental or randomized controlled trials. Protocol registered on PROSPERO (CRD42019141561). 2 reviewers screened 7 databases: SCOPUS, GEOBASE, CINAHL plus, Medline, Cochrane central register of controlled trials, EMBASE & PsycINFO from January 2000 to October 2019. Quality of included studies was assessed using the Lachowycz and Jones (2011) methodological quality assessment tool. Performed descriptive analysis.  Total no. of included studies:  14 | Zhang reviewed 14 studies and found beneficial associations between green space and reduced stress, depressive symptoms and psychological distress and with positive mood, better emotional well-being & improved mental health & behaviour. Findings were significant in 8 of the 14 studies, non-significant in 4 and inconsistent in regards to direction of effect in 2 studies.  6 of 14 studies adjusted for ethnicity.  3 studies adjusted for household SES, 1 for neighbourhood SES and 1 for neighbourhood medium income. |
| ***Cohort studies*** | | | | |  |
| **Author** | **Population**  **Setting** | **Intervention/exposure**  **Confounders adjusted for** | **Outcome**  **Follow up** | **Study design** | **Results/main findings**  **Comments on ethnicity and SES** |
| **Ahmed 2022** | Population  Children were from the MatCH study (Mothers & their Children's Health study), a sub-study of the Australian Longitudinal Study on Women's Health (general population sample). Involved 1679 children with NAPLAN Year 3 & 5 data, with mean age 8.6 years at baseline (in Year 3) & 10.6 years (Year 5).  Setting  General population observational study, with some outcome measures being undertaking in the school environment. | Intervention/exposure  Assessed for surrounding green space. Specifically assessed annual exposure to green & non-green vegetation using the Normalized Difference Vegetation Index (NDVI) and fractional cover of non-photosynthetic vegetation (fNPV), within a 100 m & 500 m buffer zone of the mother’s residential address.  Confounders  Adjusted for age, sex & socioeconomic indicators (including mother’s education, ability to manage on current income, family composition, area of residence & a formal index of relative socioeconomic advantage and disadvantage ‘IRSAD’). | Outcomes  Looked at academic outcomes & developmental vulnerability. Academic outcomes used NAPLAN data (national yearly standardized test for students, used to indicate performance in reading, writing, language & numeracy). Developmental vulnerability used AEDC data (validated national tool measuring 5 areas of childhood development at 5–6 years: physical health & wellbeing; social competence; emotional maturity; language & cognitive skills; communication skills & general knowledge).  Follow-up  Examined 2 time windows over which annual greenspace exposures was averaged: year of test (outcome measure) & year of birth to year of AEDC/NAPLAN test (approx. of child's lifetime exposure). Data on children with Year 3 & 5 NAPLAN data were analyzed longitudinally. | Study design  Cohort was a sub sample of the ‘The Australian Longitudinal Study on Women's Health’ (ALSWH) (an ongoing longitudinal study of Australian women). Women  were randomly selected from the healthcare system Medicare & surveyed every 3 years from 1996. A sample of women in 2016/17 was taken, whereby 63% of women from the 1973–78 cohort (n = 14,247) were invited to participate & complete a survey about their children in a sub-study called Mothers & their Children's Health (MatCH) study. Of those invited, 49% of mothers completed online/paper-based surveys on their 3 youngest children (n = 5799) under the ages of 13, spanning a wide range of health, development & health service use questions. Children were given an unique identifier & linked to external data, including the AEDC and NAPLAN database. | They found that exposure to non-green vegetation (fNPV) within a 500m buffer was significantly associated with academic scores at/below the NMS (national minimum standard) in year 3 and 5 for domains of reading, writing, grammar & punctuation. They found no evidence of a significant association between NDVI (measure of greenspace) & academic outcome measures in the crude/adjusted models. Despite not reaching significance, the associations were in the hypothesized direction (green space associated with better academic outcomes) for the green space measure of NDVI at 500m and 100m buffers in all academic outcome measures (except spelling) in the adjusted models.  Adjusted for socioeconomic indicators. Did not examine or adjust for ethnicity. |
| **Almeida 2022** | Population  Study included 3827 children living in predominantly urban settings (98.6%) sampled from the general population in the Porto Metropolitan areas.  Setting  Looked at surrounding green and blue space in buffer zones around the home and also the school in largely urban settings. | Intervention/exposure  Assessed for green & blue space exposure using NDVI & measures of accessibility to urban green space (UGS) & blue space in home & school surroundings, measured at 0, 4, 7, 10 years. Used buffers of 100m, 250m & 500m using the NDVI (as well as 50m when looking at schools) & buffers of 400m & 800m when looking at accessibility to UGS as well as the number of available UGS at 400 m & 800m & minimum distance to nearest UGS. Blue space assessed using Portugese Water Atlas to assess distance from children's residence & school to nearest blue space.  Confounders  Adjusted for (at age 10): sex; type of school (public/private); baseline mother's educational attainment; household monthly income; level of urbanicity; neighborhood socioeconomic deprivation; neighborhood population density. Adjusted for 2 mediators: physical activity & air pollution. | Outcomes  Verbal, performance, and global IQ, measured using WISC-III (the Wechsler Intelligence Scale for Children (administered at 10 years).  Follow-up  Green space/blue space exposure was measured at 0, 4, 7 and 10 years and outcome (verbal,  Performance & global IQ) was measured at 10 years. | Study design  Generation XXI birth cohort (G21) is a population-based birth cohort that recruited 8647 new-borns (wave 1) from 5 public maternities of the Porto Metropolitan area, between 2005- 2006. Of eligible new-borns, 91.4% of legal representatives allowed them to participate. The participants were re-evaluated at 4 years (wave 2, 2009/11, n = 7459), 7 years (wave 3, 2012/14, n = 6889) & 10 years (wave 4, 2015/17, n=6397).  Participants were excluded if they studied or lived outside the  Porto Metropolitan Area in any of the four waves or if they were not evaluated for the outcome (WISC III). | The presence of an urban green space within 800m from the home was significantly positively associated with all measures of IQ in the unadjusted model & with performance IQ (1.30 95% CI [0.26;2.35]) & global IQ (1.27 [0.18; 2.36]) in the adjusted model. This correlates to a child benefiting in regards to a 1.3% & 1.27% increase in performance & global IQ from urban green space compared to those with no urban green space in their surroundings. The authors point out that an increase of 1% of global IQ in the general population can have significant effects on economic growth of a country (quoting 2.46% and 3.80%).  Adjusted for neighbourhood socioeconomic deprivation and household income. Did not adjust for ethnicity. |
| **Anabitarte 2022** | Population  751 children aged 8 years (& later 598 of the cohort at 11–13 years), from 2 sub-cohorts of the INMA (INfancia y Medio Ambiente- Environment & Childhood) cohort study from 2 areas of Spain- Gipuzkoa & Asturias.  Setting  Populations from Asturias & Gipuzkoa (Spain), focusing on greenness surrounding the home. Asturias & Gipuzkoa are areas in North of Spain with a temperate climate with mild summers & water availability all year (have high levels of arboreal vegetation). | Intervention/exposure  Assessed greenspace exposure using 4 indicators: (1) average of NDVI (2) Vegetation Continuous Field (VCF) in buffers of 100m, 300m and 500m around the home address, (3) availability of green space within 300m from home address & (4) residential distance to green spaces.  Confounders  In cross-sectional & longitudinal analyses, adjusted for socioeconomic status, age (at time of attention test), sex, preterm, maternal IQ, maternal smoking during pregnancy & cohort. For longitudinal analysis, when models were statistically significant, they also adjusted for the mediator of NO2 (pollution). | Outcomes  Attention, measured twice at ages of 8 and 11 years, using ANT (the computerized Attentional Network Test).  Follow-up  1132 first trimester pregnant women were recruited (494 from Asturias & 638 from Gipuzkoa) in INMA. Longitudinal data up to the 8-year follow-up was available for 66% of families. At 11–13 years follow-up, data was collected from 53% of recruited families. In the case of Asturias, the notable decrease in the sample of the last follow up was due to lockdown measures (COVID-19 pandemic). | Study design  Utilized data from INMA cohort, a population-based cohort aimed at evaluating the role of environmental factors during pregnancy & childhood on the growth & development of children. The INMA cohort is conducted in 7 centres across Spain (this study is based on data from Asturias & Gipuzkoa, areas in north of Spain). | Cross-sectional analyses revealed a statistically significant protective association between average NDVI at 300m and inattentiveness (7.20, CI 95%: 13.74; 0.67) at 11-13 years. Their longitudinal analyses revealed generally beneficial associations between greenspace exposure and attention, however none were statistically significant. One key drawback may be that the green space exposure was a measure of surrounding greenspace around their homes and not in school, (which could be significant considering the time spent in school for children).  Adjusted for SES. Did not examine or adjust for ethnicity. |
| **Bloemsma 2022** | Population  3059 participants of the Dutch PIAMA birth cohort who completed the five-item Mental Health Inventory (MHI-5) at ages 11, 14, 17 and/or 20 years. General population sample from 3 different regions of the Netherlands.  Setting  Looked at residential surrounding greenness from urban & non-urban settings. | Intervention/exposure  Looked at green space, ambient air pollution & traffic noise levels at adolescents’ current home address at the times of completing the MHI-5 (i.e., recent exposures). They calculated average NDVI in buffers of 300m, 1000m & 3000m around adolescents’ homes at times of completing MHI-5, as well as using detailed land-use maps of Netherlands to assess green space.  Confounders  Assessed measures of air pollution, traffic noise exposures in conjunction with green space.  Controlled for co-variates: age, sex, parental level of education, any smoking in adolescent’s home, season & neighborhood socioeconomic status (SES). | Outcomes  Looked at mental well-being using the MHI-5. Measured this at ages 11, 14, 17 & 20 years. (MHI-5 is a widely used validated questionnaire used to assess mental wellbeing and consists of 5 questions: “How much of the time, during the last month, have you 1) been a very nervous person?; 2) felt calm & peaceful?; 3) felt downhearted & blue?; 4) been a happy person?; & 5) felt so down in the dumps that nothing could cheer you up?” Response options ranged from 1 (constantly) to 5 (never).  Follow-up  3059 participants of the Dutch PIAMA birth cohort completed the MHI-5 at ages 11, 14, 17 and/or 20 years. | Study design  Study was conducted within the ongoing Dutch Prevention & Incidence of Asthma and Mite Allergy (PIAMA) birth cohort study.  This is a cohort study where women were recruited in 1996/1997 from the general population in 3 different regions of Netherlands during their 2^nd^ trimester of pregnancy. Baseline study population consisted of 3963 children. When adolescents were 11, 14 & 17 years, parents & adolescents were requested to complete questionnaires. At age 20 years, only adolescents themselves filled in a questionnaire. | The odds of having poor mental wellbeing from ages 11 - 20 years was lower with higher average NDVI and total percentage of green space in a buffer of 3000 m (adjusted odds ratio (OR) 0.78 [95% CI 0.68–0.88] per IQR increase in the average NDVI; adjusted OR 0.77 [95% CI 0.67–0.88] per IQR increase in the total percentage of green space). These findings remained after adjusting for air pollution, traffic noise & degree of urbanization. Relationships between green space in buffers of 300m & 1000m and mental wellbeing were less clear.  Adjusted for SES. Did not examine or adjust for ethnicity. |
| **Naya 2022** | Population  Sample consisted of 202 mother–child dyads, recruited at public elementary schools with low - moderate income levels, in Los Angeles area. Approx. half of sample of children were Hispanic. Children’s age ranged from 8 - 12 years (mean 9.60 years).  Setting  Looked at residential greenness in ‘Sausage Network Buffers (SNBs)’ around child’s home address (Los Angeles area). | Intervention/exposure  Looked at residential surrounding greenness in SNBs around child’s home address. Did this by assessing park coverage within each 500m SNB using Los Angeles County Department of Parks & Recreation Countywide parks & open spaces (POS) GIS data. Also assessed levels of greenness exposure within each 500m SNB using aerial imagery from National Agriculture Imagery Programme of US Department of Agriculture (using NDVI).  Confounders  Demographic co-variates: child’s ethnicity, sex, age. Controlled for individual annual household income at baseline as well as neighborhood SES. Adjusted for mother’s baseline anxiety or depressive symptoms. | Outcomes  Looked at children’s internalizing symptoms, using subscales of the Revised Child Anxiety and Depression Scale (RCADS), (47-item child self-reported questionnaire that measures internalizing symptoms). Subscales were the six-item Generalized Anxiety Disorder subscale & the 10-item Major Depressive Disorder subscale.  Follow-up  There were 202 dyads in Wave 1 (baseline), 163 dyads in Wave 2, 151 in Wave 3, 154 in Wave 4 and 150 in Waves 5 & 6. The 6 waves were conducted over 3 years, with mean age at baseline of children being 9.6 years. | Study design  Data came from the MATCH Study- a Los Angeles-based longitudinal observational study looking into children’s health outcomes with six semi-annual assessment waves across 3 years. They used latent growth curve modelling (LGCM) to model longitudinal changes in children’s anxiety & depressive symptoms across 6 assessment waves (spanning 3 years). | They found cross-sectionally & longitudinally, that greenness exposure or park coverage was not associated with children’s depressive or anxiety symptoms. These null findings between neighbourhood greenspaces & internalising symptoms were unexpected by the author in light of previous evidence. One limitation they felt may have impacted this, was that they did not control for father and/other caregiver’s anxiety or depressive symptoms.  Adjusted for ethnicity (Hispanic or non-Hispanic) and SES. |

**Table 2: Summary results.**

| **Table 2: Summary results of the included reviews and cohort studies** | | | |
| --- | --- | --- | --- |
| **Study** | **How they have defined nature and the nature intervention** | **Level of detail in how**  **a) nature and b) the nature intervention were defined.**  Red= poor (unable to categorize into framework)  Yellow= some detail (but insufficient to categorize into framework)  Green= good (enough detail to categorize into proposed framework, with framework category detailed). | **Outcomes**  **Summary results indicated by (+) where nature is reported to have a beneficial impact, (U) where results are unclear (plus further descriptive detail providing context), and (-) for results suggesting nature may have a negative impact on mental health and well-being.** |
| ***Child population: reviews*** | | | |
| Alderton et al. 2019 | Nature & public  open space ‘i.e., publicly accessible land, such as a park, that may be used for recreation purposes’  Examined the quality, quantity or access to neighborhood public open space | a) poor | Mental health difficulties (+)  Mental health competence (+) |
|  |  | b) good: access, quantity and quality |  |
| De Keijzer et al. 2016 | 'Green space or natural environment…including parks or gardening etc’  Exposure to green space (however studies included those examining surrounding green space, views of greenness at school, activities in a green setting) | a) poor | Cognition (+) |
|  |  | b) aimed to look at exposure, but included studies that conceptualized this is different ways |  |
| Luque- Garcia et al. 2022 | 'Greenness…refers to landscapes dominated by vegetation’  Exposure to greenness (but studies actually were mostly measures of surrounding greenness, some assessed for access and some asked about use) | a) some detail but insufficient | Neuropsychological development (+)  Mental health (+) |
|  |  | b) aimed to look at exposure, but included studies that conceptualized this is different ways |  |
| Dankiw et al. 2020 | Nature was highlighted as: forest, green spaces, outdoors, gardens and natural elements  Play within nature | a) detailed but heterogeneous and unable to categorize | Cognitive development (+)  Social outcomes (+)  Emotional outcomes (+) |
|  |  | b) engagement |  |
| ***Child population: cohort studies*** | | | |
| Almeida et al. 2022 | ‘Greenness’  Exposure to greenness and accessibility to green and blue spaces in the home and school surroundings | a) poor | Performance IQ & global IQ (+) |
|  |  | b) accessibility and exposure |  |
| Anabitarte et al. 2022 | ‘Greenspace’. Defined in the introduction: ‘urban greenspaces are defined as land that is partly or completely covered with grass, trees, shrubs, or other vegetation… greenspace includes parks, community gardens, and cemeteries’  Exposure to greenspace | a) detailed but heterogeneous and unable to categorize | Attention  (+): cross-sectional evidence  (U): longitudinal evidence  Findings in direction of change to support green space exposure being beneficial, however results from longitudinal analysis did not reach significance. A significant difference was only seen at the cross-sectional analysis using a 300 m buffer around home.  Study did not control for pollution, social engagement or exercise and looked at proxy measures of exposure. |
|  |  | b) aimed to look at ‘exposure’ but did this by measures of surrounding green space and accessibility (availability) |  |
| Naya et al. 2022 | Looked at ‘park coverage’ and ‘greenness’ exposure | a) poor | Internalizing symptoms (U)  No significant associations in cross-sectional or longitudinal analyses for depressive or anxiety symptoms. Did not control for pollution (air/noise), social engagement or exercise. Used proxy measures of green space exposure. |
|  |  | b) access and exposure. (Park coverage –access to parks, and greenness exposure- used proxy measure being surrounding greenness) |  |
| ***Adolescent population: reviews*** | | | |
| Fleckney et al. 2021 | Urban public realm of which surrounding green space was a key focus. Did not define ‘green space’. Some studies looked at parks, some ‘green cover’  Didn’t pre-specify or define type of nature intervention. Some studies looked at exposure, some access, some ‘use’, some detailed quantity and quality of greenspace | a) poor | Mental health & well-being  (+): cross-sectional evidence  (U): longitudinal evidence  2 out of 4 longitudinal studies found support for nature’s beneficial effects, however significance was not discussed and these studies had moderate or high risk of bias. Of the other 2 studies, 1 found mixed results in men and women- with little positive impact in men and a negative impact in women (however significance was not reported and the study had moderate risk of bias). The final study found no evidence of an association (positive or negative). This study had moderate to high risk of bias. |
|  |  | b) not pre-defined. Detailed in some studies but not all and heterogenous |  |
| Reece et al. 2021 | ‘Green space’ in the urban environment  Exposure to nature, that was broadly defined to include any length of time exposed to green space or nature (e.g. including one-off exposures and longer-term residential exposure) | a) poor | Anxiety and depression (U)  Unable to conclude benefit, due to the lack of studies examining clinical measures of anxiety and depression. Of 201 studies, only 11 examined depression outcomes, 17 anxiety outcomes and 26 both (anxiety & depression), with the rest (n=147; 73.1%) examining ‘other’ outcomes (e.g.: mood, well-being, stress, self-esteem, quality of life). They did not report study characteristics or findings of included studies examining depression/anxiety outcomes. |
|  |  | b) aimed to look at exposure, but included studies that conceptualized this is different ways (e.g.: some involved engagement with nature, some use, some exposure…) |  |
| Zhang et al. 2020 | Green space was defined as urban areas of vegetation. In the introduction they also detail other definitions of green space  Intervention not clearly defined, but they discuss different ways of looking at green space in introduction (e.g.: accessibility, use, quantity, quality) | a) unclear | Mental health (+) |
|  |  | b) not pre-defined (and varied between individual studies) |  |
| Fang et al. 2021 | Outdoor Education Programs (OEPs) defined as: 'an intervention that included outdoor-related activities e.g.: outdoor camping, wilderness adventure, nature-based field trips, resident camp, backpacking trip | a) setting of OEP appears to be in ‘natural environments’, but not made explicit | Self-efficacy (+) |
|  |  | b) nature engagement |  |
| ***Child and adolescent populations: reviews*** | | | |
| Bikomeye et al. 2021 | ‘School yard greening’ Not pre-defined but later described majority of studies as traditional schoolyard greening interventions where outdoor school environment is changed with a combination of natural elements. In one study the greening intervention consisted of adding AstroTurf in schools’ major playground reconstructions | a) detailed but varied across studies | Socioemotional health (+) |
|  |  | b) engagement |  |
| Fyfe-Johnson et al. 2021 | Looked at ‘nature contact’ (but didn’t define what they meant by nature). In results categorized nature as gardening, green space activity (e.g.: green space not near residence or school), residential green space, school green space, greening intervention, wilderness experience, nature walk, and 'other' | a) poor | cognitive, behavioural and/mental health outcomes (+) |
|  |  | b) poor (not made explicit, and included studies were very heterogeneous) |  |
| Mann et al. 2022 | Looked at learning activities outside in nature which they called NSLOtC (nature-specific learning outside the classroom). This included practical and experiential learning activities conducted outside in school grounds and other locations such as parks, forests, residential camps/centers or on expeditions | a) gave examples, but type of nature varied amongst studies and was not always clearly defined to allow the use of the framework | personal & social development, well-being and academic progress (+) |
|  |  | b) engagement |  |
| McCormick et al. 2017 | Looked at ‘green space’. Defined green space in the introduction as: “an area of grass, trees, or other vegetation set apart for recreational or aesthetic purposes in an otherwise urban environment”  Included studies looking at multiple things e.g.: some looked at exposure, some looked at access | a) good. Human designed environment with natural elements | mental well-being, overall health and cognitive development (+) |
|  |  | b) aimed to look at access to green space, but included studies that looked at exposure as well |  |
| Mygind et al. 2019 | Examined immersion in natural environments.  Namely the Scandinavian tradition called friluftsliv (which includes concepts of, e.g. ‘outdoor life’, ‘outdoor recreation and education’ or ‘adventure recreation and education’, but with an emphasis on the experience of closeness to nature during the activity) | a) natural environments | Mental health outcomes of: self-esteem, self-efficacy, resilience, academic & cognitive performance, skill-orientated & behavioural indicators (+)  Self-concept, problem solving and mood (U)  Examining the evidence on self-concept, problem solving and mood, the evidence appears to point in the direction of nature being beneficial; however, there is a greater mixture of both null and significant positive results making us less certain |
|  |  | b) engagement |  |
| Stea et al. 2022 | 'Nature'- looked at family-based therapy taking place outdoors. This could be for example: ecotherapy, adventure therapy, outdoor rehabilitation, nature-based programs, wilderness programs, forest bathing, and/or bushcraft (as long as role of nature was intended to have therapeutic benefits) | a) examples given but not clearly defined and varied amongst studies | Family & peer relationships, adolescent behaviour, mental health, self-perceptions/self-concept, social engagement, delinquency rates & school success (+) |
|  |  | b) engagement. Some (but not all) studies included detail on quantity |  |
| Sprague et al. 2022 | Did not predefine what they meant by ‘green space’, however results indicated studies defined it in many ways. Some studies looked at distance to greenspace, some detailed type of greenspace (e.g., parks, agriculture, gardens, etc.), some detailed if and how greenspace was used (e.g., walking, playing, etc.) and some looked at frequency of use | a) not pre-defined but results included studies where some of this detail was given e.g.: garden. However, studies varied in what they were examining | Mental health & well-being (+)  Attention and behaviour (+)  Cognitive & brain outcomes (+) |
|  |  | b) some detail given but varied across included studies therefore difficult to implement framework |  |
| Tillmann et al. 2018 | Looked for an intervention that incorporated an element of nature. What this meant varied amongst studies, e.g.: some looked at ‘greenspace’, some ‘gardens’, some ‘vegetation’, some ‘wilderness therapy’.  Categorized studies based on the intervention type- some looking at exposure, some accessibility, some engagement | b) unable to use framework as definition of nature varied across included studies | Mental health outcomes: ADHD, overall mental health, stress, resilience, health-related quality of life (+)  Emotional well-being, self-esteem, depression (U)  Depression: no good quality study examined depression, of the 3 fair quality studies, 1 found significant positive results, 1 mixed results, 1 null results.  Well-being: 4 good quality studies with 2 studies reporting significant positive results, 1 study mixed results (significant positive & null results), and 1 study null results.  Self-esteem: 2 good quality studies, 1 study finding null results and other finding significant positive results when looking at greenspace use but null results looking at residential greenspace quantity. |
|  |  | b) intervention type varied amongst included studies but could be put into framework (engagement, exposure, access), however detail lacking on quantity and quality in studies |  |
| Vanaken et al. 2018 | ‘Green space’ exposure in residential or school surroundings.  Measured subjectively or objectively e.g.: remote sensing data or land cover maps or via questionnaires | a) poor- did not define what they meant by green space | Emotional & behavioural difficulties, especially hyperactivity & inattention problems (+)  Wellbeing in children or depressive symptoms in adolescents (+) |
|  |  | b) exposure (some studies looked at a proxy to exposure- surrounding green space) |  |
| Zare et al. 2022 | In introduction, introduces definition of nature as 'natural environments’ including ‘greenspace’ (defined as land that is partly or completely covered with grass, trees, shrubs, or other vegetation).  Aimed to look at green space exposure, however included studies did this in different ways (e.g.: surrounding greenness and proximity to green spaces) | a) wide definition that spans framework | Behavioral problems (+) |
|  |  | b) aimed to look at exposure. Studies mostly did this by proxy measures (surrounding greenness and proximity). A small number of studies looked at ‘use’ and quality |  |
| ***Child and adolescent population: cohort studies*** | | | |
| Ahmed et al. 2022 | In introduction, highlighted that greenspace is not consistently defined, but usually refers to land covered by vegetation (e.g.: forests, grass, parks, & gardens). Looked at greenspace in residential surroundings. Did this by looking at surrounding greenspace/vegetation and non-green vegetation as well as parkland | a) not clear in their definition used and unable to fit into framework | Childhood development & academic outcomes (U)  Direction of change was in support of nature’s beneficial effects, but did not reach significance. Used proxy measures of exposure- NDVI and fractional cover of non-photosynthetic/green vegetation (fNPV) (e.g.: sand, rock) within 100 m and 500 m buffers around the home. Did not adjust for neighbourhood socio-economic status, pollution, exercise or social engagement. |
|  |  | b) looked at surrounding greenspace which is often used as a proxy measure of ‘exposure’. They also looked at ‘access’ due to their additional measure of looking at surrounding parkland. |  |
| Bloemsa et al. 2022 | Aimed to look at residential exposure to ‘green space’ and different types of green space (i.e., urban, natural and agricultural).  Did not clearly define what they meant by green space | a) poor | Mental well-being (+) |
|  |  | b) looked at surrounding greenspace (as a proxy of exposure). Do not know whether this greenspace includes gardens or parks which could be a measure of ‘access’ |  |

**List of 139 potentially eligible studies not included in the meta-review.**

**Cross-sectional studies (n=39)**

1. Amoly E, Dadvand P, Forns J, Lopez-Vicente M, Basagana X, Julvez J, et al. Green and blue spaces and behavioral development in Barcelona schoolchildren: the BREATHE project. Environ Health Perspect. 2014;122(12):1351-8.
2. Andrusaityte S, Grazuleviciene R, Dedele A, Balseviciene B. The effect of residential greenness and city park visiting habits on preschool Children's mental and general health in Lithuania: A cross-sectional study. Int J Hyg Environ Health. 2020;223(1):142-50.
3. Bezold CP, Banay RF, Coull BA, Hart JE, James P, Kubzansky LD, et al. The Association Between Natural Environments and Depressive Symptoms in Adolescents Living in the United States. J Adolesc Health. 2018;62(4):488-95.
4. Bijnens EM, Vos S, Verheyen VV, Bruckers L, Covaci A, De Henauw S, et al. Higher surrounding green space is associated with better attention in Flemish adolescents. Environ Int. 2022;159:107016.
5. Dadvand P, Hariri S, Abbasi B, Heshmat R, Qorbani M, Motlagh ME, et al. Use of green spaces, self-satisfaction and social contacts in adolescents: A population-based CASPIAN-V study. Environ Res. 2019;168:171-7.
6. Dockx Y, Bijnens EM, Luyten L, Peusens M, Provost E, Rasking L, et al. Early life exposure to residential green space impacts cognitive functioning in children aged 4 to 6years. Environ Int. 2022;161:107094.
7. Dzhambov A, Hartig T, Markevych I, Tilov B, Dimitrova D. Urban residential greenspace and mental health in youth: Different approaches to testing multiple pathways yield different conclusions. Environ Res. 2018;160:47-59.
8. Feng X, Astell-Burt T. The Relationship between Neighbourhood Green Space and Child Mental Wellbeing Depends upon Whom You Ask: Multilevel Evidence from 3083 Children Aged 12-13 Years. Int J Environ Res Public Health. 2017;14(3):27.
9. Feng X, Astell-Burt T, Standl M, Flexeder C, Heinrich J, Markevych I. Green space quality and adolescent mental health: do personality traits matter? Environ Res. 2022;206:112591.
10. Hazlehurst MF, Muqueeth S, Wolf KL, Simmons C, Kroshus E, Tandon PS. Park access and mental health among parents and children during the COVID-19 pandemic. BMC Public Health. 2022;22(1):800.
11. Huynh Q, Craig W, Janssen I, Pickett W. Exposure to public natural space as a protective factor for emotional well-being among young people in Canada. BMC Public Health. 2013;13:407.
12. Larson LR, Barger B, Ogletree S, Torquati J, Rosenberg S, Gaither CJ, et al. Gray space and green space proximity associated with higher anxiety in youth with autism. Health Place. 2018;53:94-102.
13. Liao J, Yang S, Xia W, Peng A, Zhao J, Li Y, et al. Associations of exposure to green space with problem behaviours in preschool-aged children. Int J Epidemiol. 2020;49(3):944-53.
14. Markevych I, Tiesler CM, Fuertes E, Romanos M, Dadvand P, Nieuwenhuijsen MJ, et al. Access to urban green spaces and behavioural problems in children: Results from the GINIplus and LISAplus studies. Environ Int. 2014;71:29-35.
15. Mennis J, Li X, Meenar M, Coatsworth JD, McKeon TP, Mason MJ. Residential Greenspace and Urban Adolescent Substance Use: Exploring Interactive Effects with Peer Network Health, Sex, and Executive Function. Int J Environ Res Public Health. 2021;18(4):08.
16. Piccininni C, Michaelson V, Janssen I, Pickett W. Outdoor play and nature connectedness as potential correlates of internalized mental health symptoms among Canadian adolescents. Prev Med. 2018;112:168-75.
17. Poulain T, Sobek C, Ludwig J, Igel U, Grande G, Ott V, et al. Associations of Green Spaces and Streets in the Living Environment with Outdoor Activity, Media Use, Overweight/Obesity and Emotional Wellbeing in Children and Adolescents. Int J Environ Res Public Health. 2020;17(17):31.
18. Reuben A, Rutherford GW, James J, Razani N. Association of neighborhood parks with child health in the United States. Prev Med. 2020;141:106265.
19. Sajady M, Gower AL, McCullough M, Jordan C. More than a View: School Landscape Features Are Associated with Improved Student Adjustment. J Dev Behav Pediatr. 2020;41(6):436-42.
20. Scott JT, Kilmer RP, Wang C, Cook JR, Haber MG. Natural Environments Near Schools: Potential Benefits for Socio-Emotional and Behavioral Development in Early Childhood. Am J Community Psychol. 2018;62(3-4):419-32.
21. Sivarajah S, Smith SM, Thomas SC. Tree cover and species composition effects on academic performance of primary school students. PLoS ONE [Electronic Resource]. 2018;13(2):e0193254.
22. Tester-Jones M, White MP, Elliott LR, Weinstein N, Grellier J, Economou T, et al. Results from an 18 country cross-sectional study examining experiences of nature for people with common mental health disorders. Sci. 2020;10(1):19408.
23. Tillmann S, Clark AF, Gilliland JA. Children and Nature: Linking Accessibility of Natural Environments and Children's Health-Related Quality of Life. Int J Environ Res Public Health. 2018;15(6):25.
24. Wang P, Meng YY, Lam V, Ponce N. Green space and serious psychological distress among adults and teens: A population-based study in California. Health Place. 2019;56:184-90.
25. Ward JS, Duncan JS, Jarden A, Stewart T. The impact of children's exposure to greenspace on physical activity, cognitive development, emotional wellbeing, and ability to appraise risk. Health Place. 2016;40:44-50.
26. Zach A, Meyer N, Hendrowarsito L, Kolb S, Bolte G, Nennstiel-Ratzel U, et al. Association of sociodemographic and environmental factors with the mental health status among preschool children-Results from a cross-sectional study in Bavaria, Germany. Int J Hyg Environ Health. 2016;219(4-5):458-67.
27. Whitten T, Stevens R, Ructtinger L, Tzoumakis S, Green MJ, Laurens KR, et al. Connection to the natural environment and well-being in middle childhood. Ecopsychology. 2018;10(4):270-9.
28. van Lier LE, Utter J, Denny S, Lucassen M, Dyson B, Clark T. Home gardening and the health and well-being of adolescents. Health Promot Pract. 2017;18(1):34-43.
29. Roe JJ, Aspinall PA, Ward Thompson C. Coping with stress in deprived urban neighborhoods: What is the role of green space according to life stage? Frontiers in Psychology Vol 8 2017, ArtID 1760. 2017;8.
30. Perez-del-Pulgar C, Anguelovski I, Cole HVS, de Bont J, Connolly J, Baro F, et al. The relationship between residential proximity to outdoor play spaces and children's mental and behavioral health: The importance of neighborhood socio-economic characteristics. Environ Res. 2021;200 (no pagination).
31. Nordbo ECA, Raanaas RK, Nordh H, Aamodt G. Disentangling how the built environment relates to children's well-being: Participation in leisure activities as a mediating pathway among 8-year-olds based on the Norwegian Mother and Child Cohort Study. Health and Place. 2020;64 (no pagination).
32. Nigg C, Niessner C, Burchartz A, Woll A, Schipperijn J. The geospatial and conceptual configuration of the natural environment impacts the association with health outcomes and behavior in children and adolescents. Int J Health Geogr. 2022;21(1):9.
33. Markevych I, Tiesler CM, Fuertes E, Romanos M, Dadvand P, Nieuwenhuijsen MJ, et al. Access to urban green spaces and behavioural problems in children: Results from the GINIplus and LISAplus studies. Environ Int. 2014;71:29-35.
34. Liao J, Yang S, Xia W, Peng A, Zhao J, Li Y, et al. Associations of exposure to green space with problem behaviours in preschool-aged children. Int J Epidemiol. 2020;49(3):944-53.
35. Ezpeleta L, Navarro JB, Alonso L, de la Osa N, Ambros A, Ubalde M, et al. Greenspace exposure and obsessive-compulsive behaviors in schoolchildren. Environment and Behavior. 2022;54(5):893-916.
36. Collado S, Corraliza JA. Children's restorative experiences and self-reported environmental behaviors. Environment and Behavior. 2015;47(1):38-56.
37. Barger B, Larson LR, Ogletree S, Torquati J, Rosenberg S, Gaither CJ, et al. Tree canopy coverage predicts lower conduct problem severity in children with ASD. Journal of Mental Health Research in Intellectual Disabilities. 2020;13(1):43-61.
38. Balseviciene B, Sinkariova L, Grazuleviciene R, Andrusaityte S, Uzdanaviciute I, Dedele A, et al. Impact of residential greenness on preschool children's emotional and behavioral problems. International Journal of Environmental Research and Public Health. 2014;11(7):6757-70.
39. Amoly E, Dadvand P, Forns J, Lopez-Vicente M, Basagana X, Julvez J, et al. Green and blue spaces and behavioral development in Barcelona schoolchildren: the BREATHE project. Environ Health Perspect. 2014;122(12):1351-8.

**Cohort studies (n=20)**

1. Asta F, Michelozzi P, Cesaroni G, De Sario M, Davoli M, Porta D. Green spaces and cognitive development at age 7 years in a rome birth cohort: The mediating role of nitrogen dioxide. Environ Res. 2021;196:110358.
2. Belanger M, Gallant F, Dore I, O'Loughlin JL, Sylvestre MP, Abi Nader P, et al. Physical activity mediates the relationship between outdoor time and mental health. Prev Med Rep. 2019;16:101006.
3. Dadvand P, Nieuwenhuijsen MJ, Esnaola M, Forns J, Basagana X, Alvarez-Pedrerol M, et al. Green spaces and cognitive development in primary schoolchildren. Proc Natl Acad Sci U S A. 2015;112(26):7937-42.
4. Dadvand P, Tischer C, Estarlich M, Llop S, Dalmau-Bueno A, Lopez-Vicente M, et al. Lifelong Residential Exposure to Green Space and Attention: A Population-based Prospective Study. Environ Health Perspect. 2017;125(9):097016.
5. Franklin M, Yin X, McConnell R, Fruin S. Association of the Built Environment With Childhood Psychosocial Stress. JAMA netw. 2020;3(10):e2017634.
6. Hartley K, Perazzo J, Brokamp C, Gillespie GL, Cecil KM, LeMasters G, et al. Residential surrounding greenness and self-reported symptoms of anxiety and depression in adolescents. Environ Res. 2021;194:110628.
7. Liao J, Zhang B, Xia W, Cao Z, Zhang Y, Liang S, et al. Residential exposure to green space and early childhood neurodevelopment. Environ Int. 2019;128:70-6.
8. Madzia J, Ryan P, Yolton K, Percy Z, Newman N, LeMasters G, et al. Residential Greenspace Association with Childhood Behavioral Outcomes. Journal of Pediatrics. 2019;207:233-40.
9. Maitre L, Julvez J, Lopez-Vicente M, Warembourg C, Tamayo-Uria I, Philippat C, et al. Early-life environmental exposure determinants of child behavior in Europe: A longitudinal, population-based study. Environ Int. 2021;153:106523.
10. McEachan RRC, Yang TC, Roberts H, Pickett KE, Arseneau-Powell D, Gidlow CJ, et al. Availability, use of, and satisfaction with green space, and children's mental wellbeing at age 4 years in a multicultural, deprived, urban area: results from the Born in Bradford cohort study. Lancet Planet Health. 2018;2(6):e244-e54.
11. Putra IGNE, Astell-Burt T, Cliff DP, Vella SA, Feng X. Is prosocial behaviour a missing link between green space quality and child health-related outcomes? Social psychiatry and psychiatric epidemiology. 2022;57(4):775-89.
12. Reuben A, Arseneault L, Belsky DW, Caspi A, Fisher HL, Houts RM, et al. Residential neighborhood greenery and children's cognitive development. Soc Sci Med. 2019;230:271-9.
13. Thygesen M, Engemann K, Holst GJ, Hansen B, Geels C, Brandt J, et al. The Association between Residential Green Space in Childhood and Development of Attention Deficit Hyperactivity Disorder: A Population-Based Cohort Study. Environ Health Perspect. 2020;128(12):127011.
14. Van Aart CJC, Michels N, Sioen I, De Decker A, Bijnens EM, Janssen BG, et al. Residential landscape as a predictor of psychosocial stress in the life course from childhood to adolescence. Environ Int. 2018;120:456-63.
15. Younan D, Tuvblad C, Li L, Wu J, Lurmann F, Franklin M, et al. Environmental Determinants of Aggression in Adolescents: Role of Urban Neighborhood Greenspace. J Am Acad Child Adolesc Psychiatry. 2016;55(7):591-601.
16. Ulset V, Vitaro F, Brendgen M, Bekkhus M, Borge AI. Time spent outdoors during preschool: Links with children's cognitive and behavioral development. Journal of Environmental Psychology. 2017;52:69-80.
17. Richardson EA, Pearce J, Shortt NK, Mitchell R. The role of public and private natural space in children's social, emotional and behavioural development in Scotland: A longitudinal study. Environ Res. 2017;158:729-36.
18. Kristine E, Bocker PC, Lars A, Constantinos T, Bo MP, Jens-Christian S. Childhood exposure to green space-A novel risk-decreasing mechanism for schizophrenia? Schizophr Res. 2018;199:142-8.
19. Engemann K, Pedersen CB, Arge L, Tsirogiannis C, Mortensen PB, Svenning JC. Childhood exposure to green space - A novel risk-decreasing mechanism for schizophrenia? Schizophr Res. 2018;199:142-8.
20. Camerini AL, Albanese E, Marciano L, Corona Immunitas Research G. The impact of screen time and green time on mental health in children and adolescents during the COVID-19 pandemic. Computers in Human Behavior Reports. 2022;7:100204.

**Randomized Controlled Trials (n=2)**

1. Sobko T, Liang S, Cheng WHG, Tun HM. Impact of outdoor nature-related activities on gut microbiota, fecal serotonin, and perceived stress in preschool children: the Play&Grow randomized controlled trial. Sci. 2020;10(1):21993.
2. Zachor DA, Vardi S, Baron-Eitan S, Brodai-Meir I, Ginossar N, Ben-Itzchak E. The effectiveness of an outdoor adventure programme for young children with autism spectrum disorder: a controlled study. Developmental Medicine & Child Neurology. 2017;59(5):550-6.

**Experimental, non-randomized, controlled (n=9)**

1. Anabitarte A, Garcia-Baquero G, Andiarena A, Lertxundi N, Urbieta N, Babarro I, et al. Is Brief Exposure to Green Space in School the Best Option to Improve Attention in Children? Int J Environ Res Public Health. 2021;18(14):13.
2. Bang KS, Kim S, Song MK, Kang KI, Jeong Y. The Effects of a Health Promotion Program Using Urban Forests and Nursing Student Mentors on the Perceived and Psychological Health of Elementary School Children in Vulnerable Populations. Int J Environ Res Public Health. 2018;15(9):11.
3. Kwok SWH, Wu CST, Tong HT, Ho CN, Leung KL, Leung YCP, et al. Effects of the School-Based Integrated Health Promotion Program With Hydroponic Planting on Green Space Use and Satisfaction, Dietary Habits, and Mental Health in Early Adolescent Students: A Feasibility Quasi-Experiment. Front. 2021;9:740102.
4. Pirchio S, Passiatore Y, Panno A, Cipparone M, Carrus G. The Effects of Contact With Nature During Outdoor Environmental Education on Students' Wellbeing, Connectedness to Nature and Pro-sociality. Front Psychol. 2021;12:648458.
5. Tesler R, Plaut P, Endvelt R. The Effects of an Urban Forest Health Intervention Program on Physical Activity, Substance Abuse, Psychosomatic Symptoms, and Life Satisfaction among Adolescents. Int J Environ Res Public Health. 2018;15(10):28.
6. Jeon JY, Kim IO, Yeon PS, Shin WS. The physio-psychological effect of forest therapy programs on juvenile probationers. International Journal of Environmental Research and Public Health. 2021;18(10) (no pagination).
7. Greenwood A, Gatersleben B. Let's go outside! Environmental restoration amongst adolescents and the impact of friends and phones. Journal of Environmental Psychology. 2016;48:131-9.
8. Dettweiler U, Gerchen M, Mall C, Simon P, Kirsch P. Choice matters: Pupils' stress regulation, brain development and brain function in an outdoor education project. The British journal of educational psychology. 2022;24.
9. Amicone G, Petruccelli I, De Dominicis S, Gherardini A, Costantino V, Perucchini P, et al. Green breaks: The restorative effect of the school environment's green areas on children's cognitive performance. Frontiers in Psychology Vol 9 2018, ArtID 1579. 2018;9.

**Experimental non-randomized, no control (n=24)**

1. Bowen DJ, Neill JT, Crisp SJR. Wilderness adventure therapy effects on the mental health of youth participants. Eval Program Plann. 2016;58:49-59.
2. Chiumento A, Mukherjee I, Chandna J, Dutton C, Rahman A, Bristow K. A haven of green space: learning from a pilot pre-post evaluation of a school-based social and therapeutic horticulture intervention with children. BMC Public Health. 2018;18(1):836.
3. Frazier SL, Dinizulu SM, Rusch D, Boustani MM, Mehta TG, Reitz K. Building Resilience After School for Early Adolescents in Urban Poverty: Open Trial of Leaders @ Play. Adm Policy Ment Health. 2015;42(6):723-36.
4. Gabrielsen LE, Harper NJ, Fernee CR. What are constructive anxiety levels in wilderness therapy? An exploratory pilot study. Complement Ther Clin Pract. 2019;37:51-7.
5. Johnson EG, Davis EB, Johnson J, Pressley JD, Sawyer S, Spinazzola J. The effectiveness of trauma-informed wilderness therapy with adolescents: A pilot study. Psychol Trauma. 2020;12(8):878-87.
6. Kucher N, Larson JM, Fischer G, Mertaugh M, Peterson L, Gershan LA. 3-Dimensional nature-based therapeutics in pediatric patients with total pancreatectomy and islet auto-transplant. Complement Ther Med. 2020;48:102249.
7. Lewis SF. Examining changes in substance use and conduct problems among treatment-seeking adolescents. Child Adolesc Ment Health. 2013;18(1):33-8.
8. Machackova K, Dudik R, Zeleny J, Kolarova D, Vins Z, Riedl M. Forest Manners Exchange: Forest as a Place to Remedy Risky Behaviour of Adolescents: Mixed Methods Approach. Int J Environ Res Public Health. 2021;18(11):26.
9. Mutz M, Muller J. Mental health benefits of outdoor adventures: Results from two pilot studies. J Adolesc. 2016;49:105-14.
10. Ritchie SD, Wabano MJ, Russell K, Enosse L, Young NL. Promoting resilience and wellbeing through an outdoor intervention designed for Aboriginal adolescents. Rural Remote Health. 2014;14:2523.
11. Rocher M, Silva B, Cruz G, Bentes R, Lloret J, Ingles E. Benefits of Outdoor Sports in Blue Spaces. The Case of School Nautical Activities in Viana do Castelo. Int J Environ Res Public Health. 2020;17(22):16.
12. Song MK, Bang K-S, Kim S, Lee G, Jeong Y. Effects of an urban forest-based health promotion program on children living in group homes. Journal of Psychosocial Nursing and Mental Health Services. 2020;58(6):18-29.
13. Vankanegan C, Tucker AR, McMillion P, Gass M, Spencer L. Adventure therapy and its impact on the functioning of youth in a community setting. Social Work with Groups. 2019;42(2):127-41.
14. Tucker AR, Combs KM, Bettmann JE, Chang T-H, Graham S, Hoag M, et al. Longitudinal outcomes for youth transported to wilderness therapy programs. Research on Social Work Practice. 2018;28(4):438-51.
15. Puhakka R, Rantala O, Roslund MI, Rajaniemi J, Laitinen OH, Sinkkonen A. Greening of daycare yards with biodiverse materials affords well-being, play and environmental relationships. International Journal of Environmental Research and Public Health. 2019;16(16) (no pagination).
16. Harper N, Mott A, Obee P. Client perspectives on wilderness therapy as a component of adolescent residential treatment for problematic substance use and mental health issues. Children and Youth Services Review Vol 105 2019, ArtID 104450. 2019;105.
17. Gustafsson PE, Szczepanski A, Nelson N, Gustafsson PA. Effects of an outdoor education intervention on the mental health of schoolchildren. Journal of Adventure Education and Outdoor Learning. 2012;12(1):63-79.
18. Gabrielsen LE, Eskedal LT, Mesel T, Aasen GO, Hirte M, Kerlefsen RE, et al. The effectiveness of wilderness therapy as mental health treatment for adolescents in Norway: A mixed methods evaluation. International Journal of Adolescence and Youth. 2019;24(3):282-96.
19. Gabrielsen LE, Harper NJ, Fernee CR. What are constructive anxiety levels in wilderness therapy? An exploratory pilot study. Complement Ther Clin Pract. 2019;37:51-7.
20. Combs KM, Hoag MJ, Javorski S, Roberts SD. Adolescent self-assessment of an outdoor behavioral health program: Longitudinal outcomes and trajectories of change. Journal of Child and Family Studies. 2016;25(11):3322-30.
21. Arbuthnott KD, Sutter GC. Songwriting for nature: Increasing nature connection and well-being through musical creativity. Environmental Education Research. 2019;25(9):1300-18.
22. Acton J, Carter B. The impact of immersive outdoor activities in local woodlands on young carers emotional literacy and well-being. Comprehensive Child and Adolescent Nursing. 2016;39(2):94-106.
23. Sachs AL, Coringrato E, Sprague N, Turbyfill A, Tillema S, Litt J. Rationale, Feasibility, and Acceptability of the Meeting in Nature Together (MINT) Program: A Novel Nature-Based Social Intervention for Loneliness Reduction with Teen Parents and Their Peers. International Journal of Environmental Research and Public Health. 2022;19(17) (no pagination).
24. Schultz C, Rosen AE. School Gardens' Impact on Students' Health Outcomes in Low-Income Midwest Schools. J Sch Nurs. 2022;38(5):486-93.

**Experimental non-randomized cross-over trial (n=4)**

1. Wallner P, Kundi M, Arnberger A, Eder R, Allex B, Weitensfelder L, et al. Reloading pupils' batteries: Impact of green spaces on cognition and wellbeing. International Journal of Environmental Research and Public Health. 2018;15(6) (no pagination).
2. Stevenson MP, Dewhurst R, Schilhab T, Bentsen P. Cognitive restoration in children following exposure to nature: Evidence from the attention network task and mobile eye tracking. Frontiers in Psychology Vol 10 2019, ArtID 42. 2019;10.
3. Largo-Wight E, Guardino C, Wludyka PS, Hall KW, Wight JT, Merten JW. Nature contact at school: The impact of an outdoor classroom on children's well-being. International Journal of Environmental Health Research. 2018;28(6):653-66.
4. van den Berg AE, van den Berg CG. A comparison of children with ADHD in a natural and built setting. Child Care Health Dev. 2011;37(3):430-9.

**Qualitative study (n=10)**

1. Barfield PA, Ridder K, Hughes J, Rice-McNeil K. Get Outside! Promoting Adolescent Health through Outdoor After-School Activity. Int J Environ Res Public Health. 2021;18(14):06.
2. Chawla L, Keena K, Pevec I, Stanley E. Green schoolyards as havens from stress and resources for resilience in childhood and adolescence. Health Place. 2014;28:1-13.
3. Drake CJ, Keith M, Dober MR, Evans S, Olive LS. A qualitative investigation into the perceived therapeutic benefits and barriers of a surf therapy intervention for youth mental health. Complement Ther Med. 2021;59:102713.
4. Fernee CR, Mesel T, Andersen AJW, Gabrielsen LE. Therapy the Natural Way: A Realist Exploration of the Wilderness Therapy Treatment Process in Adolescent Mental Health Care in Norway. Qual Health Res. 2019;29(9):1358-77.
5. Lam V, Romses K, Renwick K. Exploring the Relationship between School Gardens, Food Literacy and Mental Well-Being in Youths Using Photovoice. Nutrients. 2019;11(6):16.
6. Mattouk M, Talhouk SN. A content analysis of nature photographs taken by Lebanese rural youth. PLoS ONE [Electronic Resource]. 2017;12(5):e0177079.
7. O'Brien L. Engaging with and Shaping Nature: A Nature-Based Intervention for Those with Mental Health and Behavioural Problems at the Westonbirt Arboretum in England. Int J Environ Res Public Health. 2018;15(10):10.
8. Orson CN, McGovern G, Larson RW. How challenges and peers contribute to social-emotional learning in outdoor adventure education programs. J Adolesc. 2020;81:7-18.
9. Wiens V, Kyngas H, Polkki T. The meaning of seasonal changes, nature, and animals for adolescent girls' wellbeing in northern Finland: A qualitative descriptive study. Int J Qual Stud Health Well-being. 2016;11:30160.
10. Short-Robbins SK. Therapeutic wilderness experiences and empathetic perception of at-risk adolescent youth. Dissertation Abstracts International: Section B: The Sciences and Engineering. 2021;82(5-B):No Pagination Specified.

**Narrative or rapid review (n=13)**

1. Jimenez MP, DeVille NV, Elliott EG, Schiff JE, Wilt GE, Hart JE, et al. Associations between Nature Exposure and Health: A Review of the Evidence. Int J Environ Res Public Health. 2021;18(9):30.
2. Leavell MA, Leiferman JA, Gascon M, Braddick F, Gonzalez JC, Litt JS. Nature-Based Social Prescribing in Urban Settings to Improve Social Connectedness and Mental Well-being: a Review. Curr Environ Health Rep. 2019;6(4):297-308.
3. Mainella FP, Agate JR, Clark BS. Outdoor-based play and reconnection to nature: a neglected pathway to positive youth development. New Dir Youth Dev. 2011;2011(130):89-104.
4. McCurdy LE, Winterbottom KE, Mehta SS, Roberts JR. Using nature and outdoor activity to improve children's health. Curr Probl Pediatr Adolesc Health Care. 2010;40(5):102-17.
5. Neville AR, Moothathamby N, Naganathan M, Huynh E, Moola FJ. "A place to call our own": The impact of camp experiences on the psychosocial wellbeing of children and youth affected by cancer - A narrative review. Complement Ther Clin Pract. 2019;36:18-28.
6. Owens M, Bunce HLI. The Potential for Outdoor Nature-Based Interventions in the Treatment and Prevention of Depression. Front Psychol. 2022;13:740210.
7. Ray H, Jakubec SL. Nature-based experiences and health of cancer survivors. Complement Ther Clin Pract. 2014;20(4):188-92.
8. Searight HR, Robertson K, Smith T, Perkins S, Searight BK. Complementary and alternative therapies for pediatric attention deficit hyperactivity disorder: a descriptive review. ISRN Psychiatry. 2012;2012:804127.
9. Touloumakos AK, Barrable A. Adverse Childhood Experiences: The Protective and Therapeutic Potential of Nature. Front Psychol. 2020;11:597935.
10. Wales M, Martensson F, Hoff E, Jansson M. Elevating the Role of the Outdoor Environment for Adolescent Wellbeing in Everyday Life. Front Psychol. 2022;13:774592.
11. Wray A, Martin G, Ostermeier E, Medeiros A, Little M, Reilly K, et al. Physical activity and social connectedness interventions in outdoor spaces among children and youth: a rapid review. Health Promot Chronic Dis Prev Can. 2020;40(4):104-15.
12. Pont SJ, Zaplatosch J, Lamar M, Milligan-Toffler S, Louv R, Frumkin H, et al. Green schoolyards support healthy bodies, minds and communities. Pediatrics Conference: National Conference on Education. 2017;142(1).

**Expert advise (n=1)**

1. Jameson JA. Therapeutic wilderness experiences for youth: Outdoor behavioral healthcare. Dissertation Abstracts International: Section B: The Sciences and Engineering. 2018;79(4-B(E)):No Pagination Specified.

**Case study/series (n=3)**

1. Zamani Z. Affordance of cognitive play by natural and manufactured elements and settings in preschool outdoor learning environments. Dissertation Abstracts International: Section B: The Sciences and Engineering. 2015;75(7-B(E)):No Pagination Specified.
2. Liu X. Healthy designed environments for pre-school children: Investigating ways to optimize the restoration experience in nature-based outdoor play environments. Dissertation Abstracts International Section A: Humanities and Social Sciences. 2020;81(12-A):No Pagination Specified.
3. Knibbe C, Hansen-Adamidis P. Garden as canvas: Therapeutic metaphors in a children's garden. Canadian Art Therapy Association Journal. 2017;30(1):22-30.

**Incorrect outcome (n=14)**

1. Fernee CR, Gabrielsen LE, Andersen AJ, Mesel T. Unpacking the Black Box of Wilderness Therapy: A Realist Synthesis. Qual Health Res. 2017;27(1):114-29.
2. Gass M, Wilson T, Talbot B, Tucker A, Ugianskis M, Brennan N. The Value of Outdoor Behavioral Healthcare for Adolescent Substance Users with Comorbid Conditions. Subst Abus. 2019;13:1178221819870768.
3. Kjonniksen L, Wiium N, Fjortoft I. Affordances of School Ground Environments for Physical Activity: A Case Study on 10- and 12-Year-Old Children in a Norwegian Primary School. Front. 2022;10:773323.
4. Lyons R, Colbert A, Browning M, Jakub K. Urban greenspace use among adolescents and young adults: An integrative review. Public Health Nursing. 2022;39(3):700-18.
5. Squillacioti G, Carsin AE, Bellisario V, Bono R, Garcia-Aymerich J. Multisite greenness exposure and oxidative stress in children. The potential mediating role of physical activity. Environ Res. 2022;209:112857.
6. Watchman M, Demers CMH, Potvin A. Biophilic school architecture in cold climates. Indoor and Built Environment. 2021;30(5):585-605.
7. Scott DA, Duerson LM. Continuing the discussion: A commentary on "Wilderness therapy: Ethical considerations for mental health professionals.". Child & Youth Care Forum. 2010;39(1):63-8.
8. Sachs NA, Rakow DA, Shepley MM, Peditto K. The potential correlation between nature engagement in middle childhood years and college undergraduates' nature engagement, proenvironmental attitudes, and stress. Frontiers in Psychology Vol 11 2020, ArtID 540872. 2020;11.
9. Pickett KE, Ajebon M, Hou B, Kelly B, Bird PK, Dickerson J, et al. Vulnerabilities in child well-being among primary school children: a cross-sectional study in Bradford, UK. BMJ Open. 2022;12(6) (no pagination).
10. Pearson AL, Bottomley R, Chambers T, Thornton L, Stanley J, Smith M, et al. Measuring blue space visibility and 'blue recreation' in the everyday lives of children in a capital city. International Journal of Environmental Research and Public Health. 2017;14(6) (no pagination).
11. Gabrielsen LE, Harper NJ. The role of wilderness therapy for adolescents in the face of global trends of urbanization and technification. International Journal of Adolescence and Youth. 2018;23(4):409-21.
12. Fernee CR, Gabrielsen LE, Andersen AJ, Mesel T. Emerging stories of self: Long-term outcomes of wilderness therapy in Norway. Journal of Adventure Education and Outdoor Learning. 2021;21(1):67-81.
13. Coffey JS, Gauderer L. When pediatric primary care providers prescribe nature engagement at a state park, do children "fill" the prescription? Ecopsychology. 2016;8(4):207-14.
14. Blair A, Marryat L, Frank J. How community resources mitigate the association between household poverty and the incidence of adverse childhood experiences. Int J Public Health. 2019;64(7):1059-68.

**Results of risk of bias assessment of the 16 systematic and 2 scoping reviews using AMSTAR 2 criteria. Numbers refer to questions in the AMSTAR- 2 guide and detailed below the table.**

| **AMSTAR 2 TOOL QUESTIONS:** | **1** | **2** | **3** | **4** | **5** | **6** | **7** | **8** |
| --- | --- | --- | --- | --- | --- | --- | --- | --- |
|  |  |  |  |  |  |  |  |  |
| **Alderton A, 2019** |  |  |  |  |  |  |  |  |
| **Bikomeye, 2021** |  |  |  |  |  |  |  |  |
| **Dankiw KA, 2020** |  |  |  |  |  |  |  |  |
| **Fang, B.-B., 2021** |  |  |  |  |  |  |  |  |
| **Fleckney P, 2021** |  |  |  |  |  |  |  |  |
| **Fyfe-Johnson AL, 2021** |  |  |  |  |  |  |  |  |
| **de Keijzer C, 2016** |  |  |  |  |  |  |  |  |
| **Luque-García, 2021** |  |  |  |  |  |  |  |  |
| **Mann J, 2022** |  |  |  |  |  |  |  |  |
| **McCormick R, 2017** |  |  |  |  |  |  |  |  |
| **Mygind, 2019** |  |  |  |  |  |  |  |  |
| **Reece R, 2021** |  |  |  |  |  |  |  |  |
| **Stea T.H, 2022** |  |  |  |  |  |  |  |  |
| **Sprague N.L, 2022** |  |  |  |  |  |  |  |  |
| **Tillmann S, 2018** |  |  |  |  |  |  |  |  |
| **Vanaken GJ, 2018** |  |  |  |  |  |  |  |  |
| **Zare Sakhvidi MJ, 2022** |  |  |  |  |  |  |  |  |
| **Zhang Y, 2020** |  |  |  |  |  |  |  |  |

| **AMSTAR 2 TOOL**  **QUESTIONS** | **9** | | **10** | **11** | | **12** | **13** | **14** | **15** | **16** |
| --- | --- | --- | --- | --- | --- | --- | --- | --- | --- | --- |
|  | **RCTs** | **NSRI** |  | **RCTs** | **NSRI** |  |  |  |  |  |
| **Alderton A, 2019** | n/a |  |  | n/a | n/a | n/a |  |  | n/a |  |
| **Bikomeye, 2021** |  | n/a |  | n/a | n/a | n/a |  |  | n/a |  |
| **Dankiw KA, 2020** | n/a |  |  | n/a | n/a | n/a |  |  | n/a |  |
| **Fang, B.-B., 2021** |  |  |  |  |  |  |  |  |  |  |
| **Fleckney P, 2021** | n/a |  |  | n/a | n/a | n/a |  |  | n/a |  |
| **Fyfe-Johnson AL, 2021** |  |  |  | n/a | n/a | n/a |  |  | n/a |  |
| **de Keijzer C, 2016** | n/a |  |  | n/a | n/a | n/a |  |  | n/a |  |
| **Luque-García, 2021** | n/a |  |  | n/a | n/a | n/a |  |  | n/a |  |
| **Mann J, 2022** |  |  |  | n/a | n/a | n/a |  |  | n/a |  |
| **McCormick R, 2017** |  |  |  | n/a | n/a | n/a |  |  | n/a |  |
| **Mygind, 2019** |  |  |  | n/a | n/a | n/a |  |  | n/a |  |
| **Reece R, 2021** | n/a |  |  | n/a | n/a | n/a |  |  | n/a |  |
| **Stea T.H, 2022** | n/a |  |  | n/a | n/a | n/a |  |  | n/a |  |
| **Sprague N.L, 2022** |  |  |  | n/a | n/a | n/a |  |  | n/a |  |
| **Tillmann S, 2018** |  |  |  | n/a | n/a | n/a |  |  | n/a |  |
| **Vanaken GJ, 2018** |  |  |  | n/a | n/a | n/a |  |  | n/a |  |
| **Zare Sakhvidi MJ, 2022** | n/a |  |  | n/a | n/a | n/a |  |  | n/a |  |
| **Zhang Y, 2020** |  |  |  | n/a | n/a | n/a |  |  | n/a |  |

**AMSTAR 2 Tool colour key: Red- no, orange- partial yes, green- yes, grey- N/A.**

**AMSTAR 2 Tool question key:**

1. Did the research questions and inclusion criteria for the review include the components on the PICO?
2. Did the report of the review contain an explicit statement that the review methods were established prior to conduct of the review and did the report justify any significant deviations from the protocol?
3. Did the review authors explain their selection of the study designs for inclusion in the review?
4. Did the review authors use a comprehensive literature search strategy?
5. Did the review authors perform study selection in duplicate?
6. Did the review authors perform data extraction in duplicate?
7. Did the review authors provide a list of excluded studies and justify the exclusions?
8. Did the review authors describe the included studies in adequate detail?
9. Did the review authors use a satisfactory technique for assessing the risk of bias (RoB) in individual studies that were included in the review? For RCTS (randomized controlled studies) and for NRSI (non-randomized studies of interventions).
10. Did the review authors report on the sources of funding for the studies included in the review?
11. If meta-analysis was justified did the review authors use appropriate methods for statistical combination of results? For RCTs and for NRSIs.
12. If meta-analysis was performed did the review authors assess the potential impact of RoB in individual studies on the results of the meta-analysis or other evidence synthesis?
13. Did the review authors account for RoB in individual studies when interpreting/ discussing the results of the review?
14. Did the review authors provide a satisfactory explanation for, and discussion of, any heterogeneity observed in the results of the review?
15. If they performed quantitative synthesis did the review authors carry out an adequate investigation of publication bias (small study bias) and discuss its likely impact on the results of the review?
16. Did the review authors report any potential sources of conflict of interest, including any funding they received for conducting the review?

**Results of risk of bias assessment of the cohort studies published after the date of the last review, using the adapted Newcastle- Ottawa Scale (NOS) risk of bias tool for cohort studies. Numbers refer to questions in the NOS tool and detailed below the table.**

| **Cohort studies-** | **SELECTION** | |  |  | **COMPARABILITY** | **OUTCOME** |  |  | TOTAL STARS | RATING |
| --- | --- | --- | --- | --- | --- | --- | --- | --- | --- | --- |
|  | **1** | **2** | **3** | **4** | **5** | **6** | **7** | **8** |  |  |
|  | **Represent-ativeness** | **selection** | **exposure** | **start** | **comparability** | Assessment | follow-up | adequacy |  |  |
| **Almeida 2022** | x | x | x |  | x | x | x | x | 7 | Good |
| **Anabitarte 2022** | x | x | x |  | x | x | x |  | 6 | good |
| **Bloemsma 2022** | x | x | x |  | x | x | x | X | 6 | good |
| **Maitre 2021** | x | x | x |  |  | x | x |  | 5 | poor |
| **Putra 2022** | x | x |  |  |  | x | x |  | 4 | poor |
| **Naya 2022** | x | x | x |  | x | x | x |  | 6 | good |
| **Ahmed 2022** | x | x | x |  | x | x | x | x | 7 | good |
| **Camerini 2022** | x | x |  |  | x | x |  | x | 5 | fair |

**Newcastle- Ottawa Scale (NOS) adapted for Cohort studies.**

**Key:**

Green- low risk of bias

red- high risk of bias.

X= star awarded (low risk of bias), identified as * below. Note: some sections can be awarded up to 2 stars.

*Selection:*

1. Representativeness of the exposed cohort:
   1. Truly representative *
   2. Somewhat representative *
   3. Selected group
   4. No description of the derivation of the cohort
2. Selection of the non-exposed cohort:
   1. Drawn from the same community as the exposed cohort *
   2. Drawn from a different source
   3. No description of the derivation of the non-exposed cohort
3. Ascertainment of exposure:
   1. Secure/objective record (e.g.: NDVI, GPS mapping) *
   2. Structured interview *
   3. Written self-report
   4. No description
   5. Other
4. Demonstration that outcome of interest was not present at start of study:
   1. Yes *
   2. No

*Comparability:*

1. Comparability of cohorts on the basis of the design or analysis controlled for confounders.
   1. The study controls for age, sex, socioeconomic status*
   2. Study controls for other factors: pollution, noise, social contact/engagement and exercise*
   3. Cohorts are not comparable on basis of design/analysis did not control for confounders

*Outcome:*

1. Assessment of outcome:
2. Independent blind assessment using objective validated tests **
3. Unblinded (or blinding not reported) assessment using objective validated tests *
   1. Used non-standard or non-validated tests
   2. Self-report
   3. Other
   4. No description
4. Was follow-up long enough for outcomes to occur:
   1. Yes * (1 year minimum – felt suitable time frame to assess long term/sustainment of mental health effects of green space exposure considering neuroplasticity etc)
   2. No
5. Adequacy of follow-up cohorts
   1. Complete follow-up – all subjects accounted for *
   2. Subjects lost to follow-up unlikely to introduce bias- number lost less than or equal to 20% or description of those lost suggested no different from those followed *
   3. Follow up rate less than 80% and no description of those lost
   4. No statement

Scoring

*Good quality:* 3 or 4 stars in selection domain AND 1 or 2 in comparability domain AND 2 or 3 stars in outcome/exposure domain

*Fair quality:* 2 stars in selection domain AND 1 or 2 in comparability domain AND 2 or 3 stars in outcome/exposure domain

*Poor quality:* 0 or 1 stars in selection domain OR 0 stars in comparability domain OR 0 or 1 stars in outcome/exposure domain

**Results of risk of bias assessment of the cross-sectional studies published after the date of the last review, using the adapted Newcastle- Ottawa Scale (NOS) risk of bias tool. Numbers refer to questions in the NOS tool and detailed below the table.**

| **Cross-sectional** | **SELECTION** | |  |  | **COMPARABILITY** | **OUTCOME** |  | TOTAL STARS | RATING |
| --- | --- | --- | --- | --- | --- | --- | --- | --- | --- |
|  | **1** | **2** | **3** | **4** | **5** | **6** | **7** |  |  |
|  | **Represent-ativeness** | **sample size** | **non-respondents** | **exposure** | **comparability** | Assessment | statistical test | |  |
| **Bijnens 2022** | x |  |  | x | x | x | x | 5 | fair |
| **Dockx 2022** | x |  |  | x | x | x | x | 5 | fair |
| **Feng 2022** | x |  | x |  | x | x | x | 5 | fair |
| **Hazlehurst 2022** | x |  |  |  |  | x | x | 3 | poor |
| **mennis 2021** |  |  |  | x | x | x | x | 4 | poor |
| **Perez-del-Pulger 2021** | x |  |  | x | x | xx | x | 6 | fair |
| **Nigg 2022** | x |  |  | x | x | xx | x | 6 | fair |
| **Ezpeleta 2022** | x |  | x | x | x | x | x | 6 | fair |

**Newcastle- Ottawa Scale (NOS) adapted for cross-sectional studies.**

**Key:**

Green- low risk of bias

red- high risk of bias.

X= star awarded (low risk of bias), identified as * below. Note: some sections can be awarded up to 2 stars.

*Selection:*

1. Representativeness of the sample:
   1. Truly representative of the average in the target population * (all subjects or random sampling)
   2. Somewhat representative of the average in the target group * (non-random sampling)
   3. Selected group of users/convenience sample
   4. No description of the derivation of the included subjects
2. Sample size:
   1. Justified and satisfactory (including sample size calculation) *
   2. Not justified
   3. No information provided
3. Non-respondents:
   1. Proportion of target sample recruited attains pre-specified target or basic summary of non-respondent characteristics in sampling frame recorded *
   2. Unsatisfactory recruitment rate, no summary data on non-respondents
   3. No information provided
4. Ascertainment of the exposure (risk factor):
   1. Objective measure of surrounding nature/green space e.g.: NDVI or GPS/mapping (accessing access/availability/proxy to exposure) and measure of green space use (e.g.: self-report of use) **
   2. Objective measure of surrounding nature/green space e.g.: NDVI or GPS/mapping (accessing access/availability/proxy to exposure) only*
   3. Parental/personal recall only.

*Comparability:* *(Maximum 2 stars)*

1. Comparability of subjects in different outcome groups on the basis of design or analysis. Confounding factors controlled.
   1. Data/results adjusted for age, sex, socioeconomic status *
   2. Data/results adjusted for pollution, noise, social contact/engagement and exercise*
   3. Data/results not adjusted for all relevant confounders/risk factors/information not provided.

*Outcome: (Maximum 2 stars)*

1. Assessment of outcome:
   1. Independent blind assessment using objective validated tests **
   2. Unblinded assessment using objective validated tests *
   3. Used non-standard or non-validated tests
   4. No description/non-standard methods used
2. Statistical test:
   1. Statistical test used to analyse the data clearly described, appropriate and measures of association presented including confidence intervals and probability level (p value) *
   2. Statistical test not appropriate, not described or incomplete

Cross-sectional Studies scoring:

Good Studies: 8-10 stars

Fair Studies: 5-7 stars

Poor Studies: 0 to 4 stars

This scale has been adapted from the Newcastle-Ottawa Quality Assessment Scale for cohort studies to provide quality assessment of cross-sectional studies.

**Results of risk of bias assessment of the experimental studies (all were non-randomized) published after the date of the last review, using the ROBINS-I risk of bias tool. Details of the ROBINS-I tool can be found below the table.**

| **Non-randomized experimental studies - ROBINS-I** | | | |  |  |  |  |  |
| --- | --- | --- | --- | --- | --- | --- | --- | --- |
|  | **Confounding** | **Selection** | **classification** | **deviations** | **missing data** | **outcomes** | **reporting** | **overall bias** |
| **Jeon 2021** |  |  |  |  |  |  |  | moderate |
| **Anabitarte 2021** | 4 |  |  |  |  |  |  | moderate |
| **Kwok 2021** |  |  |  |  |  |  |  | serious |
| **Machackova 2021** |  |  |  |  |  |  |  | serious |
| **Pirchio 2021** |  |  |  |  |  |  |  | serious |
| **Dettweiler 2022** |  |  |  |  |  |  |  | moderate |
| **Sachs 2022** |  |  |  |  |  |  |  | moderate |
| **Scultz 2022** |  |  |  |  |  |  |  | serious |

**Risk Of Bias In Non-randomized Studies – of Interventions (ROBINS-I) tool.**

**Key:**

Blue: low risk of bias

Yellow: moderate risk of bias

Orange: serious risk of bias

Red: critical risk of bias

Grey: no information

Pre-specified list of confounding domains relevant to all or most studies: Exercise, social contact, noise, pollution.

Pre-specified list of co-interventions that could be different between intervention groups and that could impact on outcomes: Exercise, social contact, counselling/psychological therapy, training/skills acquisition, mentoring/couching.

For full quality assessment tool, see our protocol on the OSF website (searching the papers title), however key domains were (highlighted in the table headings):

- Bias due to confounding
- Bias in selection of participants into the study
- Bias in classification of interventions
- Bias due to deviations from intended interventions
- Bias due to missing data
- Bias in measurement of outcomes
- Bias in selection of the reported result
- Overall bias
